# High abundance of antimicrobial resistance genes in chicken flocks receiving antimicrobial treatment in Vietnamese poultry production

**DOI:** 10.1093/jacamr/dlaf117

**Published:** 2025-07-04

**Authors:** Avijit Dutta, Bach Tuan Kiet, Nguyen Thi Nhung, Ellen Higginson, Leanne Kermack, Nguyen Thi Phuong Yen, Doan Hoang Phu, Marc Choisy, Juan Carrique-Mas, Stephen Baker

**Affiliations:** Cambridge Institute for Therapeutic Immunology and Infectious Disease, University of Cambridge, Cambridge, UK; Department of Microbiology and Veterinary Public Health, Chattogram Veterinary and Animal Sciences University, Chattogram 4225, Bangladesh; Sub-Department of Animal Health and Production, Dong Thap Province, Cao Lanh, Vietnam; Oxford University Clinical Research Unit, Ho Chi Minh City, Vietnam; Parasites and Microbes Programme, Welcome Sanger Institute, Cambridge, UK; Cambridge Institute for Therapeutic Immunology and Infectious Disease, University of Cambridge, Cambridge, UK; Oxford University Clinical Research Unit, Ho Chi Minh City, Vietnam; Oxford University Clinical Research Unit, Ho Chi Minh City, Vietnam; Faculty of Animal Science and Veterinary Medicine, Nong Lam University, Ho Chi Minh City, Vietnam; Oxford University Clinical Research Unit, Ho Chi Minh City, Vietnam; Centre for Tropical Medicine and Global Health, University of Oxford, Oxford, UK; Food and Agriculture Organization of the United Nations (FAO), Hanoi, Vietnam; A*STAR Infectious Diseases Labs (A*STAR IDL), Agency for Science, Technology and Research (A*STAR), Singapore 138648, Singapore

## Abstract

**Background and objectives:**

Studies focussing on measuring antimicrobial resistance (AMR) rely on phenotyping or low throughput PCR detection of limited AMR genes (ARGs); high-throughput qPCR (HT-qPCR) may be a scalable approach for measuring AMR. We applied Fluidigm HT-qPCR to measure the impact of flock-level antimicrobial use (AMU) on genotypic AMR in the Mekong Delta area of Vietnam.

**Methods:**

AMU-related data and pooled faecal samples were collected longitudinally from 20 meat chicken flocks, divided into flocks treated with antimicrobials and untreated controls. Samples were analysed for 94 ARGs using Fluidigm HT-qPCR. Normalized ARG abundance was measured in reference to 16S rRNA. A regression model was constructed to weigh the effect of AMU factors on AMR.

**Results:**

The frequency of ARGs per sample was significantly higher in antimicrobial treatment group chicken samples (56.4; 95% CI 55.3–57.6) compared with the controls (52.1, 95% CI 50.9–53.4). Similarly, the normalized ARG abundance was significantly greater in treatment flock samples (3.2; 95% CI 2.9–3.4) than in control samples (2.0; 95% CI 1.7–2.3), except for tetracycline ARGs. Overall, ARG frequency negatively correlated with the average ARG abundance (*R* = −0.27 and *P* < 0.05). The time series analysis of ARG abundance revealed three distinct and equally prevalent patterns of ARG persistence. Among all the AMU factors, the number of antimicrobial classes and the total AMU duration showed the highest impact on flock ARG abundance.

**Conclusion:**

The findings of this study highlight the utility of molecular AMR profiling in areas with heavy AMU for poultry production.

## Introduction

Antimicrobial resistance (AMR) is currently a major global health challenge. Approximately 700 000 deaths per year are predicted to be attributed to AMR globally, with an estimated economic burden of $100 trillion by 2050.^1,2^ The burden of AMR is particularly high in low- and middle-income countries (LMICs), which is likely associated with excessive antimicrobial use (AMU) coupled with limited capacity for infection control and a lack of prevention facilities and resources.^3^

Vietnam is considered an AMR hotspot due to a high incidence of communicable diseases, and unregulated access to antimicrobials for human, animal health, and livestock production.^4,5^ Nevertheless, in response to the WHO’s Global Action Plan on AMR, Vietnam became the first country in the WHO’s Western Pacific Region to develop its National AMR Action Plan (2013–2020).^6^ This plan addressed key issues such as hospital-based AMR surveillance, improvement of infection control measures, restriction to antimicrobial access, and public awareness for the safe use of antimicrobials in human and veterinary sectors.^7^ Subsequently, the Vietnamese Ministry of Agriculture and Rural Development issued a separate action plan to fight against AMR in livestock and aquaculture (2017–20).^8^ One of the stimuli to take these initiatives was that approximately three-quarters (72%) of AMU in the country occurs in livestock production and is exceptionally high in chicken production.^9,10^ Additionally, the use of antimicrobials as growth promoters has been banned in Vietnam since 2018 (Circular 6/2016/TT-BNNPTNT).^11^

AMU is an important selective force for AMR; therefore, a dynamic relationship between antimicrobial exposure and AMR development is rational.^12^ In chicken production, factors like frequency, dose and duration of AMU, bird age, number and combination of antimicrobial therapies, farm husbandry and biosecurity practices play interdependent roles in shaping the flock AMR.^13,14^ Furthermore, age-dependent host microbiota composition, pre-existing resistome, and antimicrobial residues may also affect this AMU-AMR relationship.^15,16^ Nevertheless, some studies have reported a direct significant association between AMU and both phenotypic and genotypic AMR in poultry independent of other factors mentioned.^17,18^ A large-scale metagenomic study of broiler farms across nine European countries identified a significant positive correlation between AMU and their corresponding AMR genes (ARGs).^19^ Yet, AMU is not the only risk factor determining AMR in livestock, and therefore, the demonstration of an association between AMU and AMR is not straightforward.^20^

Several studies have established a high degree of AMU and AMR in chicken production in the Mekong Delta of Vietnam.^21,22^ However, the majority of AMR studies have been conducted exploiting one or a few bacterial species and their phenotypic resistance characterization or detection of a limited number of ARGs using conventional PCR.^14,23^ This approach results in limited data regarding resistance mechanism(s) and ARG abundance. Here, by employing an HT-qPCR technique using a comprehensive panel of ARGs, we prospectively investigated the impact of farm-level AMU on the genotypic AMR patterns and ARG abundance in chicken flocks of the Mekong Delta, Vietnam. Using time series analysis of ARG abundance, we studied the persistence of ARG and also measured how different AMU-related farm factors contribute to flock AMR.

## Methods

### Ethics

The study’s ethical approval was obtained from the Oxford University Ethics Committee (OxTREC, Ref No. 5121/16). The poultry farmers participating in the study gave written informed consent.

### Study design, sample collection and DNA extraction

We recruited chicken flocks from 20 farms raising native flocks located in four districts of Dong Thap province of the Mekong Delta, Vietnam, raising >100 birds in an all-in-all-out system. Farmers were provided with a record book organized by week and were requested to record the farm production and management data. Before antimicrobial administration, ten birds were randomly selected and raised separately from the treatment flock and were denominated as the control group receiving no antimicrobials. The treatment flock, when required, received antimicrobials mostly through the water. The project coordinator visited the farm prior to AMU to collect samples. A descriptive summary of the farm-level AMU is mentioned in Table S1 (available as Supplementary data at *JAC-AMR* Online).

Pooled chicken faecal samples from both control and treatment groups of each farm were collected at the following time points: before starting any antimicrobial treatment to the respective flock, after the conclusion of the flock’s first round of treatment by specific antimicrobial class, on Days 7, 14, 30, 60 and 90 of postantimicrobial treatment, and right before depopulation (Figure S1). Samples were collected using sterile paper liners (0.5 m × 0.5 m), placed near drinkers or feeders in the chicken pen in the evening, and the deposited droppings were collected the next morning. Liners were swabbed using sterile gauze. Each of the collected swabs was placed in a universal container and mixed vigorously with 50 mL of buffer saline. Ultimately, 1 mL of the resultant eluate was stored at −20°C with glycerol until extraction.

From the collected samples, total DNA was extracted using a QIAamp DNA Mini Kit (Qiagen, Germany), and concentration was measured using Nanodrop (Thermo Fisher, USA). For detailed analysis, the extracted DNA was shipped to the Cambridge Institute for Therapeutic Immunology and Infectious Disease, University of Cambridge, UK.

### ARG assay panel

A comprehensive list of 94 ARGs conferring resistance to commonly used antimicrobials in poultry production, including ESBL and vancomycin resistance, was designed for the study (Table S2). To assess the bacterial biomass and normalise the ARG abundance, a 16S rRNA marker was added. For positive control purposes, synthetic plasmids with the vector pUC57 were mapped using Geneious Prime (www.geneious.com/prime).

### Fluidigm HT-qPCR and data curation

HT-qPCR was performed using a 96.96 Biomark^TM^ Dynamic Array for Real-Time PCR system (Standard BioTools, USA). Firstly, diluted sample DNA (1.25 μL) was subjected to preamplification using Bio-Rad T100 PCR Thermocycler (Bio-Rad Laboratories, USA) with the conditions: 95°C for 2 minutes, followed by 12 cycles of 95°C for 15 seconds and 60°C for 4 minutes. After cleaning up, the amplicon was diluted 5-fold. Pre-amplified DNA and assays were loaded into a 96.96 integrated fluidic circuit. Final thermal cycling and real-time imaging were performed on the BioMark HD following the manufacturer’s instructions. The plasmid DNA was used as a positive control, and a sample without template DNA was used as a negative control. The final thresholding of the results was conducted based on the average melting temperature (Tm) of all samples having Ct ≤ 20 ± 1°C.

### Normalized ARG abundance calculation

The normalized ARG abundance was calculated compared with the 16S rRNA using the formula described elsewhere.^24^ The Ratio _(test ARG/16s rRNA)_ = 2^−ΔCt^ where ΔCt = Ct_ARG—_Ct_16S rRNA_. Within the control and treatment flock, normalized abundance data of all other sampling points were standardized in reference to the first time point. All the ARG abundance data were log-transformed (log2), visualized and analysed accordingly.

### ARG abundance time series analysis

We analysed the correlation between the time series of ARG abundance using a similarity network method.^25^ First, we computed the correlation (Spearman’s rank correlation) between all pairwise time series of ARG abundance. A correlation was considered statistically significant if the correlation coefficient (*ρ*) was >0.8 and the *P* value was <0.01.^26^ The *P* values were adjusted to reduce the chance of false positivity following the Benjamini-Hochberg multiple correlation method.^27^ Statistically significant pairwise correlations among ARGs were given as inputs to construct and visualize a similarity network using Gephi.^28^

### Statistical analysis

The cumulative ARG/16S rRNA abundance was summarized according to antimicrobial class and sampling time points and was compared between the control and treatment flocks using the unpaired t-test. The prevalence of individual ARGs with a 95% CI was calculated using the Modified Wald Method, and the comparison of samples for an ARG (present/absent) in the control and treatment flocks was made by the Chi-square test. Correlation analysis was employed to measure the relationship between the frequency and the abundance of ARG.

Univariable linear regression analysis was conducted to unveil the effect of AMU-related farm factors such as frequency, duration and interval of AMU, age of the bird and number and combination of the antimicrobial classes on ARG abundance using SPSS. Control flock samples without any AMU were used as the reference.

## Results

### Study flocks and AMU

A total of 261 samples were collected from 20 poultry flocks. The average flock duration was 97 days (95% CI 87.2–106.8). The chickens received antimicrobial treatment at different ages, ranging from day-old chicks (DOC) to as old as 87 days. The antimicrobials used in the treatment flocks, individually or in combination, were beta-lactams, aminoglycosides, Macrolide-Lincosamide-Streptogramin B (MLS_B_), tetracyclines, sulphonamides, polymyxins, quinolones, and phenicols. The average duration of AMU was 14.9 days (95% CI 8.9–20.9), ranging from 2 to 38 days. Four flocks (20%) had AMU for >30 days each. Twelve out of 20 farms (60%) had at least two courses of AMU over the whole production cycle (25% farms: three courses and 15% farms: four courses).

### ARG frequency and diversity

On average, irrespective of sampling time points, the treatment flock chicken samples recorded significantly higher ARGs/sample (56.4; 95% CI 55.3–57.6) compared with the control group (52.1; 95% CI 50.9–53.4) (*P* < 0.05) (Figure S2). Again, within the treatment flock, the mean ARG frequency of 57.8 (95% CI 56.8–58.7) for samples collected post-first round antimicrobial treatment (‘after’) was significantly higher than the samples collected before any antimicrobial exposure (48.7; 95% CI 44.5–52.8) (*P* < 0.05). Notably, for the control group samples, the difference between these two sampling points was less pronounced. Out of a total of 94 ARGs tested, 74 and 76 unique ARGs were detected in the control and treatment flocks, respectively. Among 11 classes of antimicrobials investigated, ∼60% of the total ARG diversity (control 59.5% and treatment 57.9%) fell into three major categories: multidrug, beta-lactams, and aminoglycosides ARGs (Table S3).

### Shift in normalized ARG abundance

Prior to antimicrobial administration, there was no significant difference in ARG abundance between the treatment and control flock samples, with an overall 4.5% normalized ARG abundance difference (*P* = 0.739) (Table 1). After antimicrobial exposure, there was a significant upsurge (58.5%) in the abundance of ARGs in the treatment flock samples (*P* = 0.003). Notably, on Day 7, a 29.2% reduction in the cumulative normalized abundance in the treatment flock samples was observed (*P* = 0.033). For the last two sampling points of the treatment flock samples, Day 90 and ‘end’, there was a rise in ARG abundance belonging to genes associated with resistance to beta-lactams (degree of difference, Day 90, 173.2; end 770.6) and polymyxins (205.4 versus 523.8). At the end of the production cycle, treatment flock samples had an overall 14.7% ARG abundance increase (before 19.0; end 21.8), whereas the control flock sample recorded an 18.7% decline (before 18.2, end 14.8).

**Table 1.** Antimicrobial class-wise cumulative ARG abundance difference between treatment and control groups in each sample at different sampling time points

Antimicrobial classes	Per sample cumulative ARGs/16S rRNA abundance (log2 value) (95% CI)							
	Before	After	Day 7	Day 14	Day 30	Day 60	Day 90	End
**All ARGs**								
Control	18.2 (13.9, 22.5)	12.5 (9.1, 15.9)	17.2 (13.4, 21.1)	14.6 (12.1, 17.1)	8.9 (6.5, 11.3)	9.4 (6.7, 12.0)	7.8 (4.8, 10.9)	14.8 (11.3, 18.4)
Treatment	19.0 (16.4, 21.7)	19.8 (16.5, 23.1)	12.2 (9.5, 14.9)	16.3 (13.5, 19.0)	13.6 (11.0, 16.3)	16.8 (14.1, 19.5)	17.8 (14.5, 21.1)	21.8 (17.2, 26.4)
Degree of Difference (%)	4.5	58.5	−29.2	11.3	52.7	79.1	127.4	46.6
*P* value	0.739	0.003	0.033	0.384	0.010	<0.001	<0.001	0.024
**Aminoglycoside**								
Control	49.4 (31.2, 67.7)	29.4 (14.6, 44.2)	42.4 (23.5, 61.3)	35.7 (26.7, 44.7)	22.3 (13.3, 31.1)	33.5 (22.1, 44.9)	28.6 (15.7, 41.4)	39.5 (28.0, 51.1)
Treatment	43.1 (35.2, 51.0)	49.8 (37.2, 62.4)	35.8 (23.8, 47.8)	45.1 (36.6, 53.6)	41.2 (34.0, 48.3)	52.0 (45.7, 58.2)	52.4 (43.4, 61.5)	62.4 (46.0, 78.9)
Degree of Difference (%)	−12.8	69.5	−15.5	26.4	84.8	55.2	83.6	57.9
*P* value	0.427	0.033	0.541	0.118	0.001	0.005	0.003	0.021
**Beta-lactam**								
Control	9.2 (−1.4, 20.0)	3.4 (−7.5, 14.2)	11.1 (1.3, 20.9)	5.8 (0.2, 11.5)	−7.0 (−14.2, 0.1)	−5.6 (−11.5, 0.3)	−8.1 (−15.2, −1.0)	1.7 (−5.2, 8.6)
Treatment	11.6 (6.6, 16.5)	18.1 (8.5, 27.7)	4.9 (−1.3, 11.1)	7.2 (−0.5, 14.9)	2.9 (−3.0, 8.8)	6.3 (1.6, 11.1)	5.9 (−2.1, 14.0)	14.9 (6.4, 23.3)
Degree of Difference (%)	25.3	436.2	−55.9	23.2	141.5	213.8	173.2	770.6
*P* value	0.626	0.039	0.266	0.768	0.030	0.002	0.009	0.015
**MLS_B_**								
Control	25.6 (15.3, 35.8)	22.1 (11.7, 32.6)	28.8 (18.7, 38.9)	28.3 (22.8, 33.7)	22.3 (18.2, 26.5)	22.1 (16.2, 27.9)	23.3 (14.7–31.9)	27.7 (20.0, 35.4)
Treatment	27.1 (20.6, 33.6)	27.0 (19.8, 34.2)	22.2 (15.4, 28.9)	27.3 (21.3, 33.3)	25.5 (20.7, 30.4)	25.3 (20.8, 29.8)	26.3 (20.6, 32.0)	35.5 (25.5, 45.6)
Degree of Difference (%)	6.1	22.0	−23.0	−3.4	14.3	14.8	12.6	28.2
*P* value	0.774	0.424	0.257	0.806	0.296	0.378	0.544	0.185
**Multidrug**								
Control	31.7 (13.3–50.0)	25.3 (7.5, 43.0)	29.6 (12.4, 46.7)	22.4 (13.1, 31.7)	18.2 (6.8, 29.6)	9.1 (−1.0, 19.2)	8.5 (−6.1, 23.0)	30.3 (19.6, 41.1)
Treatment	39.2 (29.2, 49.2)	40.3 (27.3, 53.3)	25.3 (14.4, 36.2)	33.1 (21.9, 44.3)	30.3 (20.5, 40.2)	35.6 (27.8, 43.5)	40.7 (32.6, 48.8)	49.4 (36.0, 62.7)
Degree of Difference (%)	24.0	59.6	−14.5	47.5	66.6	293.1	231.2	62.6
*P* value	0.399	0.158	0.659	0.133	0.101	<0.001	<0.001	0.023
**Phenicol**								
Control	−0.3 (−5.8, 5.2)	−0.4 (−2.3, 1.6)	0.2 (−2.8, 3.1)	0.3 (−0.9, 1.5)	−2.5 (−4.0, −0.9)	−0.8 (−1.7, 0.2)	−0.8 (−2.4, 0.9)	0.4 (−5.7, 6.5)
Treatment	2.6 (1.3, 4.0)	−0.4 (−2.2, 1.5)	−2.1 (−3.4, −0.7)	−0.5 (−1.6, 0.6)	−3.0 (−4.4, −1.5)	−0.5 (−1.9, 0.9)	−0.0 (−2.1, 2.1)	0.4 (−2.4, 3.2)
Degree of Difference (%)	863.9	−3.4	−1278.6	−268.9	20.7	33.6	98.3	−7.3
*P* value	0.094	0.992	0.121	0.283	0.606	0.746	0.529	0.989
**Polymyxin**								
Control	2.5 (−1.3, 6.3)	1.4 (−1.6, 4.5)	2.4 (−0.6, 5.4)	1.8 (0.3, 3.2)	1.1 (−1.1, 3.3)	−2.0 (−3.6, −0.5)	−2.1 (−4.0, −0.3)	0.7 (−0.6, 2.0)
Treatment	4.7 (2.7, 6.7)	4.2 (2.1, 6.2)	1.2 (−0.5, 2.9)	2.1 (−0.2, 4.3)	1.1 (−0.9, 3.1)	2.1 (0.8, 3.3)	2.2 (0.6, 38)	4.5 (1.9, 7.0)
Degree of Difference (%)	88.9	188.2	−48.8	18.5	−2.6	200.6	205.4	523.8
*P* value	0.226	0.120	0.473	0.805	0.984	<0.001	<0.001	0.010
**Quinoline**								
Control	1.5 (−8.2, 11.2)	−1.5 (−3.9, 1.0)	−1.2 (−4.7, 2.3)	−2.8 (−5.5, 0.0)	−3.1 (−7.0, 0.7)	−3.6 (−5.5, −1.6)	−4.1 (−7.8, −0.4)	−0.8 (−3.2, 1.7)
Treatment	0.6 (−1.6, 2.8)	−0.9 (−3.9, 2.1)	−3.5 (−6.1, −1.0)	−1.1 (−3.7, 1.5)	−1.2 (−2.6, 0.2)	−1.3 (−3.1, 0.5)	0.1 (−2.1, 2.2)	−0.3 (−2.0, 1.3)
Degree of Difference (%)	−59.9	38.2	−201.2	61.0	62.3	63.3	102.0	58.0
*P* value	0.716	0.753	0.228	0.365	0.233	0.107	0.030	0.722
**Sulfonamide**								
Control	14.8 (9.4, 20.2)	8.2 (0.7, 15.8)	11.1 (2.4, 19.7)	9.3 (5.7, 12.9)	7.6 (4.0, 11.2)	7.5 (4.0, 10.9)	4.9 (−1.8, 11.5)	10.9 (4.5, 17.4)
Treatment	15.0 (11.9, 18.2)	16.2 (10.8, 21.7)	10.8 (6.8, 14.8)	14.0 (10.3, 17.8)	12.7 (9.2, 16.1)	14.7 (11.9, 17.6)	15.4 (12.7, 18.1)	18.9 (13.7, 24.2)
Degree of Difference (%)	1.8	97.1	−2.6	50.9	67.1	97.7	215.6	73.5
*P* value	0.925	0.079	0.949	0.063	0.040	0.002	0.004	0.040
**Tetracycline**								
Control	19.5 (10.6, 28.4)	22.4 (13.3, 31.5)	24.5 (16.3, 32.6)	24.2 (19.3, 29.0)	17.1 (12.0, 22.1)	19.6 (14.3, 25.0)	19.1 (12.5, 25.7)	20.4 (14.4, 26.5)
Treatment	20.8 (15.6, 25.9)	22.0 (16.0, 27.9)	13.4 (6.7, 20.1)	22.9 (19.5, 26.3)	18.3 (12.9, 23.7)	20.3 (15.8, 24.9)	24.3 (19.0, 29.7)	24.8 (17.8, 31.8)
Degree of Difference (%)	6.5	−2.1	−45.2	−5.5	7.3	3.7	27.5	21.4
*P* value	0.776	0.928	0.034	0.640	0.721	0.829	0.194	0.303
**Glycopeptide**								
Control	0 (0)	−1.1 (0)	0 (0)	0 (0)	0 (0)	0 (0)	−0.3 (0)	−0.4 (−1.4, 0.6)
Treatment	0 (0)	−4.2 (0)	0 (0)	−0.2 (−0.3, −0.1)	−0.2 (−1.2, 0.8)	−0.3	−0.2 (0)	−0.4 (−0.6, −0.2)
Degree of Difference (%)	N/C	−277.3	N/C	N/C	N/C	N/C	1300	−1.0
*P* value	N/C	N/C	N/C	0.011	0.123	N/C	N/C	0.966
**Other**								
Control	7.6 (4.4, 10.8)	6.2 (1.9, 10.5)	7.7 (4.2, 11.2)	6.8 (3.9, 9.7)	4.4 (2.4, 6.4)	4.3 (2.1, 6.6)	3.2 (0.5, 5.9)	6.6 (4.2, 8.9)
Treatment	8.4 (6.4, 10.5)	10.4 (7.2, 13.6)	7.3 (4.8, 9.8)	8.5 (6.6, 10.4)	6.8 (4.9, 8.6)	7.8 (6.3, 9.3)	7.3 (4.7, 9.8)	10.6 (7.7, 13.5)
Degree of Difference (%)	10.8	66.9	−5.3	25.5	53.9	79.8	129.1	61.5
*P* value	0.628	0.111	0.843	0.303	0.077	0.010	0.025	0.026

N/C, not calculated.

**Figure 1.**
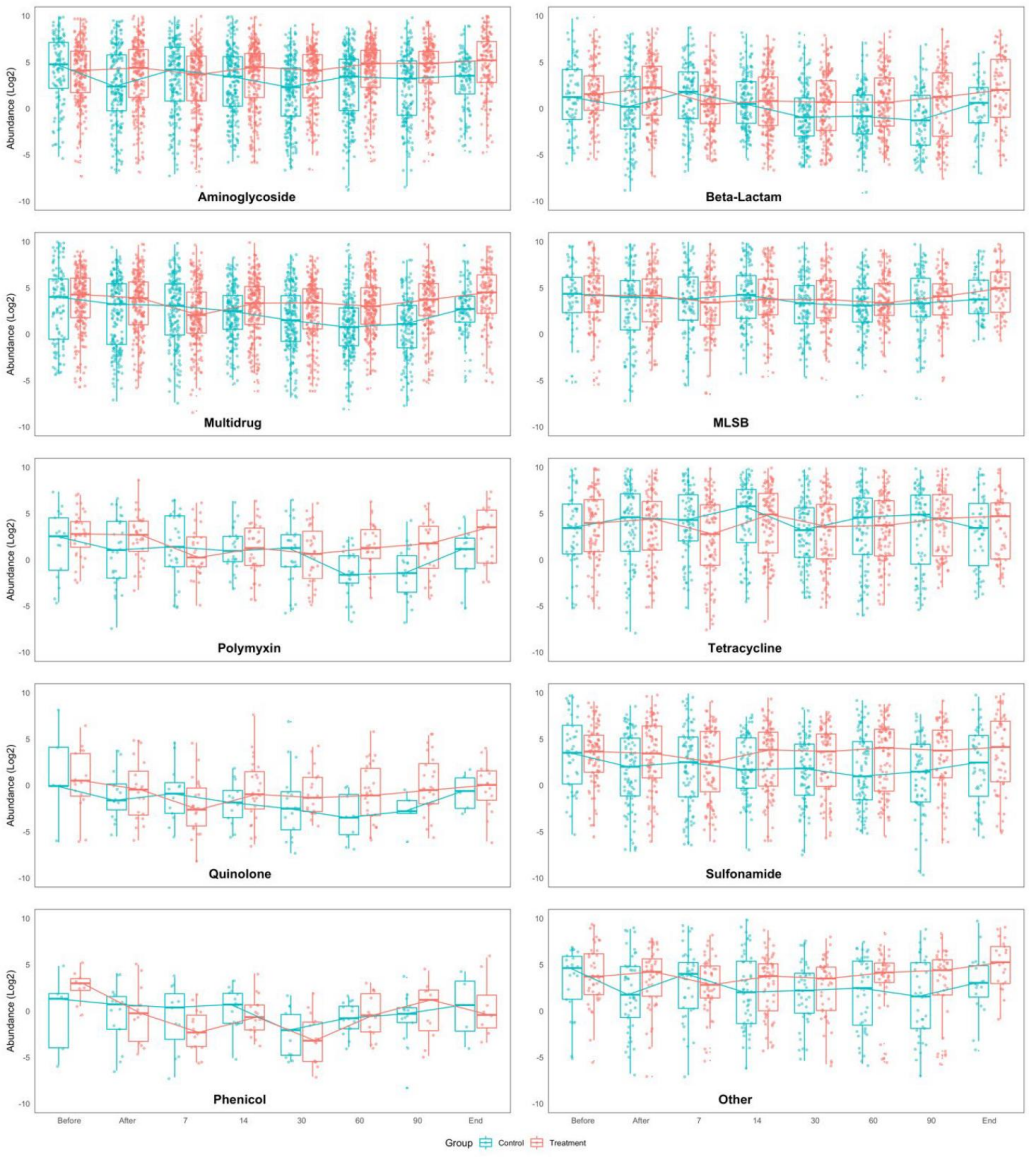
The antimicrobial class-wise average ARG/16S rRNA abundance at different sampling time points between control and treatment flocks. Each dot represents the ARGs/16S rRNA value of all ARGs corresponding to respective antimicrobial classes. Here, the log2 transformed values were used to calculate the cumulative abundance.

In the samples collected after antimicrobial administration (‘after’ sampling point), the mean normalized ARG abundance against all antimicrobial classes, except for tetracyclines, was significantly higher in the treatment flocks (3.2, 95% CI 3.1–3.5) than the control group (2.2, 95% CI 2.0–2.5) (*P* < 0.001) (Figure 1 and Table S4). On Day 7, irrespective of antimicrobial classes, the average normalized ARG abundance in the control group samples (3.1; 95% CI 2.8–3.4) significantly surpassed those of the treatment flock (2.1, 95% CI 1.8–2.3) (*P* < 0.001). For most of the antimicrobial classes, on Day 14, the average ARG abundance was higher in the treatment flock samples (mean 2.7, 95% CI 2.5–2.9) than in the control flock ones (2.6, 95% CI 2.4–2.8), except for tetracyclines (treatment versus control 4.1; 95% CI 3.4–4.9 versus 5.0; 95% CI 4.1–5.8), MLS_B_ (4.0; 95% CI 3.5–4.5 versus 4.5; 95% CI 3.9–5.1) and phenicols (−0.5; 95% CI −1.7–0.6 versus 0.3; 95% CI −0.9–1.5). For the subsequent sampling points of Day 30, Day 60, Day 90 and end, a general trend of higher mean ARG richness was observed in the treatment group samples except for tetracyclines, phenicols and polymyxins.

A significant change in the ARG abundance across different sampling time points of the treatment flock samples was observed for selected antimicrobial classes, namely, aminoglycoside, beta-lactam, phenicol and polymyxin (Table S5).

### Individual ARG abundance and prevalence

Between the control and treatment flock samples, irrespective of sampling points, we observed varying degrees of differences in the overall individual ARG abundance (Figure S3 and Table S6). The highest 3.4% abundance difference was observed for *bla_PSE_* (treatment 3.1, control −0.3; *P* < 0.001). Other ARGs with a high richness included *cfr2*, *aac(3′)-Ii(acde)*, *aac(6′)-Ib*, *bla_DHA_*, *sul3* and *mcr-1*. Some ARGs had an overall lower normalized abundance in the treatment flock samples than that of the control flock, such as *bla_GES,_ lnu(A)-01*, *dfrA*, *aac(6′)-Ii*, *oqxB*, *vanB* and *cepA2*. A prevalence analysis revealed that some ARGs were 100% present irrespective of flock type and sampling points, such as *aac(6’)-aph(2′′), aadE, aph(3′)-III, sat4, ermB*, *tetM, tetO, tetW* and *floR* (Table S6). For the majority of ARGs, there was an increase in the overall prevalence in the treatment flock samples compared with the control ones.

### Correlation between ARG frequency and abundance

**Figure 2.**
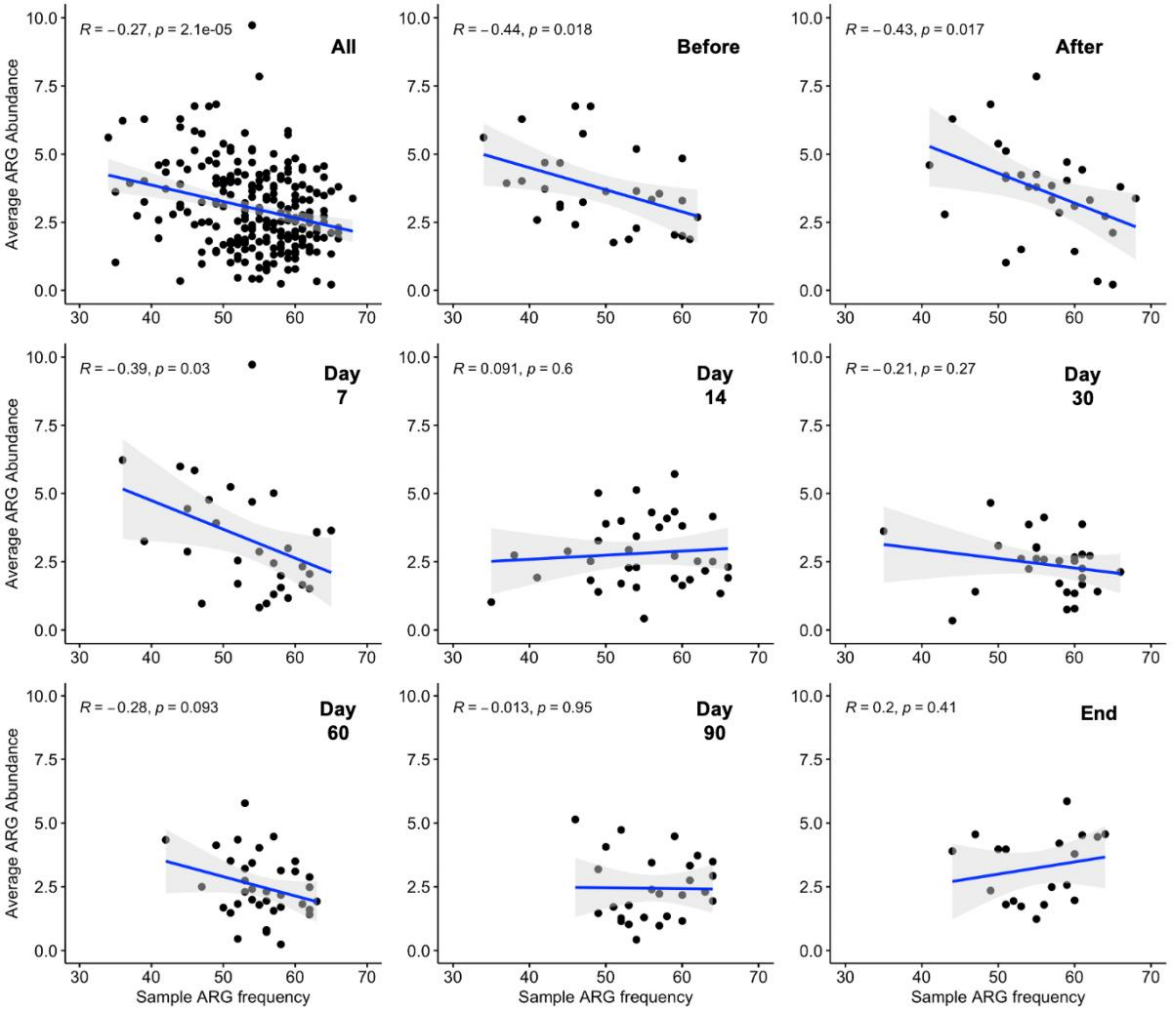
Correlation between the frequency of ARGs per sample and the average abundance of ARGs at different sampling points. Each dot represents a sample. Here, log2 values were calculated for abundance comparison among the ARGs. Pearson’s correlation coefficient was used to calculate the *R* values and a *P* value <0.05 was considered significant. A negative correlation coefficient value indicates that the abundance of resistance genes decreases with the increase in the frequency of ARGs in a sample.

Overall, irrespective of the chicken flock type and sampling point, a significant negative correlation was observed between the ARG frequency and abundance (*R* = −0.27 and *P* < 0.05) (Figure 2). However, the magnitude of the correlation varied across the sampling points. Except for a weak positive correlation on Day 14 (*R* = 0.09, *P* = 0.6) and at the end of production (*R* = 0.20, *P* *=* 0.41), for all other sampling time points, an increase in the ARG frequency of a sample was coupled with the reduction of average normalized ARG abundance.

A correlation analysis among the ARGs conferring resistance to different antimicrobial classes revealed a strong positive correlation (Figure S4). Aminoglycosides and β-lactams ARGs showed a significant positive correlation with all other classes, especially with MLS_B_, tetracyclines, sulphonamides and multi-drug ARGs (*r* = 0.3 to 0.7; *P* < 0.001). ARGs conferring resistance to multiple classes of antimicrobials displayed a significantly strong positive correlation with many other classes such as β-lactams (*r* = 0.6), polymyxins (0.6), quinolone (0.5), sulphonamides (0.5), aminoglycosides (0.4), MLS_B_ (0.4) and tetracyclines (0.4) (all *P* < 0.001). A weak negative correlation was observed between ARGs encoding resistance to phenicols and quinolones (*r* = −0.02).

### Temporal dynamics of ARG abundance

**Figure 3.**
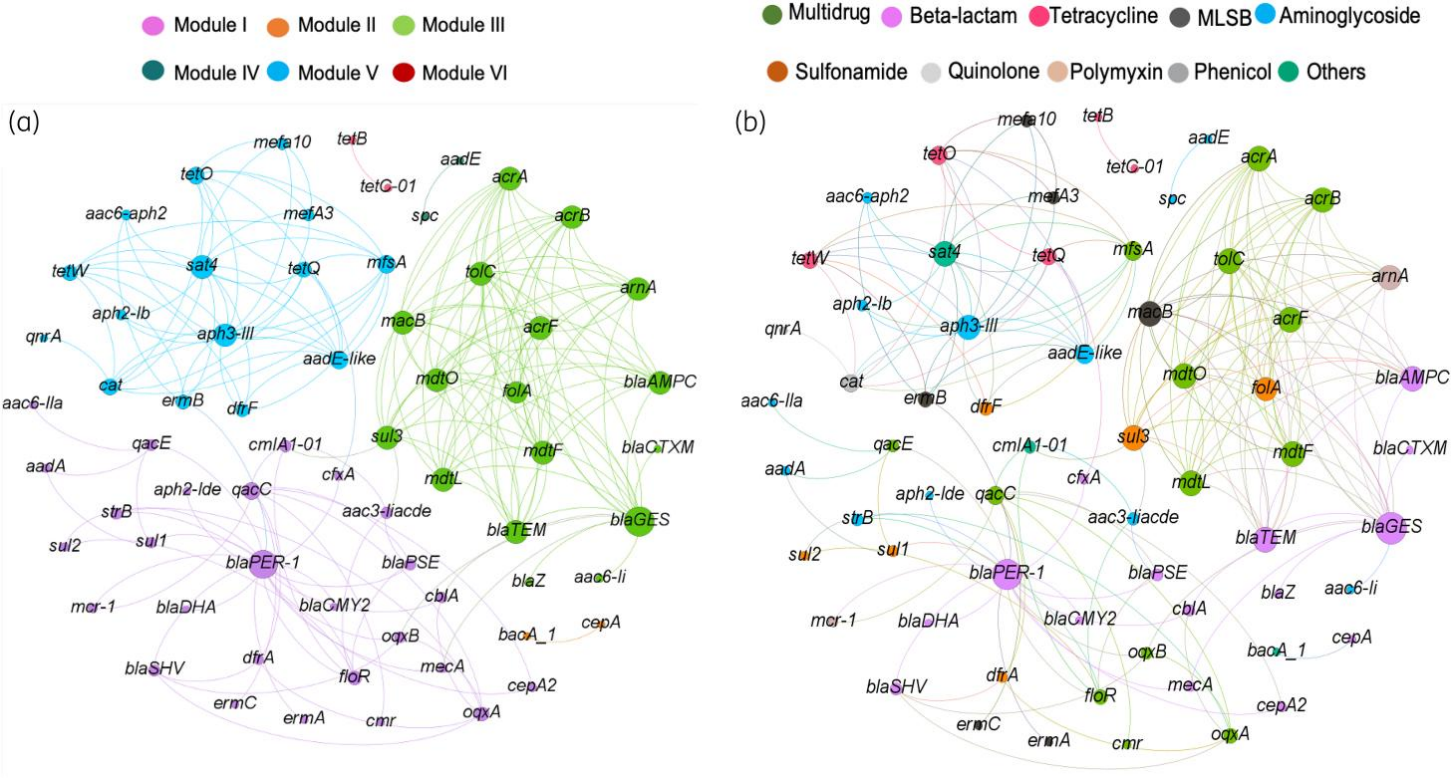
The network analysis depicting the temporal dynamics of ARG abundance among the resistance gene variants. The colour code for each node was based on modularity class (a) and ARG subtypes (b). A connection between two nodes reveals a strong significant correlation (Spearman’s correlation coefficient *ρ*>0.8 and *P* value <0.05). The node size represents connections.

**Figure 4.**
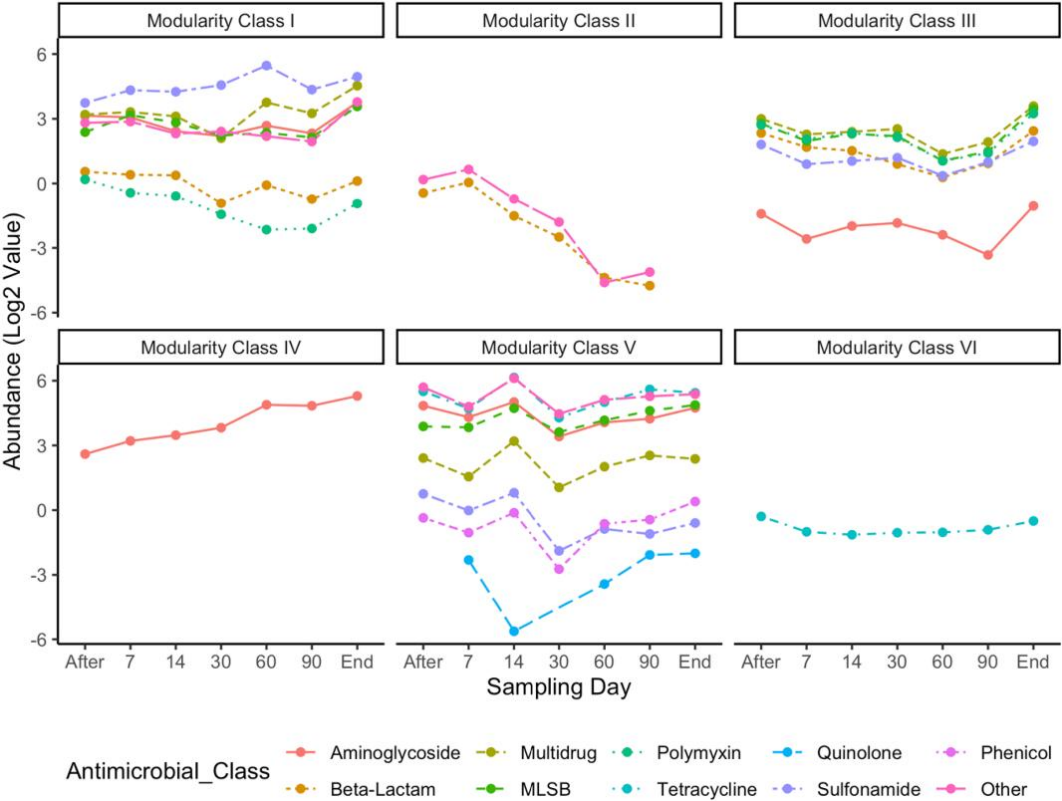
The time series analysis of ARGs of different modularity classes obtained from network analysis. Here, each dot represents the mean abundance of all the ARGs of a particular antimicrobial group within the modularity class. The data showed a temporal pattern based on different sampling time points. Here, the antimicrobial gene classes of modularity Class II and Class IV showed a downward and upward trend of ARG abundance shift, respectively, whereas Class VI remained steady over the study period. A trend was not observed for the rest of the modularity classes ARGs (Class I, Class III and Class V).

An ARG network analysis, combining both control and treatment flock ARG abundance data, revealed distinct patterns of ARG concentration dynamics similarities (Figure 3). The network plot consisted of 65 nodes (ARGs) and 194 edges. The ARG subtypes of different antimicrobial classes formed six discrete modules or clusters (i.e. aggregation of nodes or ARGs that had similar abundance dynamics over time), indicating the underpinning nature of ARG persistence. Module I, Module II and Module III consisted of 41.6%, 26.2% and 23.1% of the total connections of the network. ARG clusters with a great number of ARGs were Module I, Module III and Module V, having 27, 17 and 15 ARGs, respectively. The most densely connected node among all the clusters of the network plot, known as a hub, was a beta-lactamase, ‘*bla_GES_*’. The number of ARGs of different antimicrobial classes in each modularity class varied widely (Table S7). A time series plot of each modularity class-based ARG type revealed distinct patterns of ARG persistence, at least for three different modules (Figure 4). For modularity Class II ARGs (*cepA* and *bacA_1*), a trend of gradual abundance reduction was observed over time. Conversely, Class IV ARGs of the aminoglycoside category (*spc* and *aadE*) demonstrated a regular increasing pattern, whereas ARGs in module VI showed a plateau for tetracycline ARGs (*tetB* and *tetC-01*). A regular pattern was not observed for the rest of the modularity classes ARGs.

### AMU-related farm factors affecting AMR

The frequency of AMU showed a significant impact on the ARG abundance (*F* = 5.0, *P* = 0.001), explaining 8.2% of the ARG abundance differences between the flocks (Table 2). The total AMU duration and the interval between two consecutive AMUs were responsible for 6.5% and 8.7% of the ARG variance, respectively. In addition, the age of the chicken receiving antimicrobials in the treatment flock explained 7.4% of the total ARG abundance difference (*F* = 6.0, *P* = 0.001). There was an overall significant effect of the number of antimicrobial classes farmers administered on ARG abundance (*F* = 3.9; *R^2^* = 0.08; *P* = 0.002). Finally, the combination of different classes of antimicrobials accounted for 10.6% of ARG abundance variance (*F* = 1.8; *P* = 0.036). A combination of five antimicrobial classes namely tetracyclines, sulfonamides, polymyxins, aminoglycoside and MLS_B_ yielded the highest ARG abundance (*β* = 107.8, *t* = 2.6, *P* = 0.010).

**Table 2.** Results of univariable linear regression analysis stating the effect of AMU-related farm factors on ARG abundance

AMU-related farm variables and co-variables	Mean normalized ARG abundance (95% CI)	Regression coefficient, β (95% CI)	*P* value
**Frequency of AMU**			
No AMU	111.8 (91.2–132.3)	111.8 (93.7–129.9)	Reference
Single AMU	165.3 (140.1–190.4)	53.5 (19.8–87.0)	0.002
Two times AMU	183.9 (136.7–231.0)	72.1 (26.9–117.3)	0.002
Three times AMU	123.8 (97.0–150.6)	12.0 (−28.9–52.9)	0.564
Four times AMU	178.2 (145.8–210.6)	66.4 (17.2–115.6)	0.008
**Total AMU duration**			
No AMU	111.8 (91.2–132.3)	111.8 (93.6–130.0)	Reference
<10 days	152.5 (129.6–175.5)	40.8 (9.8–71.7)	0.010
11− 30 days	181.0 (145.9–216.1)	69.2 (29.1–109.3)	0.001
>30 days	156.3 (131.8–180.8)	44.5 (0.6–88.4)	0.047
**Interval between two subsequent AMU**			
No AMU	111.8 (91.2–132.3)	111.8 (93.6–130.0)	Reference
1 day	131.1 (41.3–220.9)	19.3 (−71.3–109.9)	0.674
5 days	126.3 (4.8–247.7)	14.5 (−95.6–124.6)	0.795
13 days	229.5 (160.8–298.1)	117.7 (52.0–183.3)	0.001
**Age of bird receiving antimicrobials**			
No AMU	111.8 (91.2–132.3)	111.8 (93.6–129.9)	Reference
AMU in the first week	173.4 (147.4–199.4)	61.6 (26.5–96.7)	0.001
AMU in first 4 weeks	181.5 (139.2–223.8)	69.7 (26.1–113.4)	0.002
AMU beyond 4 weeks	139.7 (117.9–161.6)	28.0 (−5.2–61.2)	0.098
**No of antimicrobial classes administered**			
No AMU	111.8 (91.2–132.3)	111.8 (93.6–129.9)	Reference
One antimicrobial class	186.7 (165.0–208.3)	74.9 (32.5–117.2)	0.001
Two antimicrobial classes	164.4 (125.9–202.9)	52.6 (14.1–91.1)	0.008
Three antimicrobial classes	135.1 (113.0–157.2)	23.3 (−22.1–68.6)	0.313
Four antimicrobial classes	137.0 (95.3–178.7)	25.2 (−20.1–70.6)	0.274
Five antimicrobial classes	185.3 9126.5–244.1)	73.5 (14.4–132.6)	0.015
**Combination of antimicrobial classes**			
No antimicrobial	111.8 (91.2–132.3)	111.8 (93.5–130.1)	Reference
Quinolone	194.7 (162.0–227.4)	83.0 (25.6–140.3)	0.005
Tetracycline	178.6 (146.0–211.1)	66.8 (9.4–124.1)	0.023
Tetracycline + MLS_B_	195.5 (78.4–312.5)	83.7 (1.6–165.8)	0.046
Tetracycline + polymyxin	164.5 (73.1–255.9)	52.7 (−9.1–114.6)	0.094
Tetracycline + sulfonamide	178.9 (103.4–254.4)	67.1 (−15.0–149.2)	0.108
Tetracycline + sulfonamide + MLS_B_	126.3 (4.8–247.7)	14.5 (−85.2–114.2)	0.775
Tetracycline + polymyxin + phenicol	141.6 (86.2–197.0)	29.8 (−52.3–111.9)	0.475
Tetracycline + sulfonamide + polymyxin	134.7 (107.5–162.0)	23.0 (−36.5–82.4)	0.448
Tetracycline + sulfonamide + polymyxin + MLS_B_	165.1 (113.5–216.8)	53.3 (−6.1–112.8)	0.079
Tetracycline + polymyxin + MLS_B_ + beta-lactam	131.1 (41.3–220.9)	19.3 (−62.8–101.4)	0.644
Tetracycline + sulfonamide + polymyxin + aminoglycoside + phenicol	151.0 (91.6–210.4)	39.3 (−42.8–121.4)	0.347
Tetracycline + sulfonamide + polymyxin + aminoglycoside + MLS_B_	219.5 (100.0–338.5)	107.8 (25.7–189.9)	0.010
Beta-lactam + aminoglycoside	137.0 (65.4–208.2)	25.0 (−39.6–89.7)	0.446

A forward stepwise variable selection procedure resulted in two models describing 2.7% and 6.2% ARG variations, respectively (Table 3). In Model 1, except for the number of antimicrobial classes used in the treatment chicken flock, all other variables were excluded. In Model 2, in addition to the number of antimicrobial classes, the total AMU duration was included. Both the number of antimicrobial classes and the duration of AMU showed a significant impact on ARG abundance (*P* = 0.002). Variables excluded from each model with their statistics, are mentioned elsewhere (Table S8).

**Table 3.** Results of multivariable linear regression analysis showing the summary of forward modelling

Regression model	Predictors retained in the model	Unstandardized co-efficient (*β*) (95% CI)	*t* value	*P* value	*F* statistics (degree of freedom, residual)	*R* square value (adjusted)
Model 1	No AMU	129.9 (116.0–143.7)	18.4	Reference	7.4 (1, 228)	0.027
	1 antimicrobial class	56.8 (15.5–98.1)	2.7	0.007		
Model 2	No AMU	121.0 (106.3–135.8)	16.1	Reference	8.5 (2, 227)	0.062
	1 antimicrobial class	65.6 (24.7–106.5)	3.1	0.002		
	AMU for 11 to 30 days duration	60.0 (21.5–98.5)	3.1	0.002		

## Discussion

The impact of AMU on AMR has been studied extensively in different settings, with the standard hypothesis that the administration of antimicrobials leads to increased ARG contamination. In this study, by exploiting a panel of 94 ARGs and HT-qPCR, we measured the impact of farm-level AMU on AMR in Vietnamese poultry flocks, a system typical for many LMICs. Here, we noted that the ARGs/sample and the normalized ARG abundance of the treatment flock chicken samples were significantly higher than the control ones, with the marked transition happening after the antimicrobial administration. Therefore, our data suggests that antimicrobial selection pressure on the host microbiota leads to the enrichment of ARGs.^29,30^ Here, the emergence of ARGs of certain antimicrobial classes was associated with the exposure of that respective antimicrobial on the farm. To exemplify, farms with polymyxin AMU showed a higher occurrence and abundance of *mcr-1*. An extensive AMR study in nine European nations’ broiler farms revealed a strong correlation between farm AMU and faecal resistome.^19^ Nevertheless, some ARGs (e.g. *aac(3′)-Ii(acde), aac(6′)-aph(2′′), aac(6′)-Ib, aadA* and *aadE*) were ubiquitous irrespective of the farm’s AMU profile. Additionally, samples collected before AMU also showed ARG contamination. In poultry production systems, DOC can act as a source of antimicrobial-resistant bacteria.^31^ In this study, data on the DOC source were unavailable. Nevertheless, the possible sources of the high level of AMR in the baseline samples might be a high AMU in the original parent or chick stock. Besides, the poultry farm environment, a reservoir of ARGs and AMR bacteria, may play a critical role.^32^

Emergence of AMR without apparent AMU has been reported. Bacterial genetic mutations in certain environments can occasionally co-select a resistance mutation.^33^ Furthermore, there is enough scientific evidence suggesting that day-old-chick can carry a compendium of ARGs.^34^

On Day 7, a higher normalized ARG abundance was noted in the control group samples compared with the treatment ones. We observed that the Ct value of 16S rRNA was notably high on Day 7 for treatment flock samples. The administration of antimicrobial(s) reduces the burden of susceptible bacteria whilst concurrently increasing the abundance of ARGs in resistant bacteria as they survive. The dynamic shift in the bacterial biomass and ARG is time-dependent since antimicrobial exposure-dependent selection pressure, causing resistance phenotypes, changes with time.^35,36^ Different resistance mechanisms persist for different time duration.^37,38^ Therefore, in many cases, even in the absence of AMU, the proliferation of resistant bacteria and horizontal gene transfer may predispose to observing higher normalized ARG abundance.

After a general trend of ARG abundance increase, at the end of the production cycle, for most of the ARGs, the abundance dropped. The transient multiple-fold ARG abundance increase in the first phase of production gets reduced in due course and becomes masked due to fitness cost.^35,36^ As predicted, a faster reduction rate in the control flock samples can be attributed to the absence of direct AMU.

Among all the ARGs investigated, multi-drug ARGs predominated both in control and treatment flocks, followed by beta-lactams and aminoglycosides. The multi-drug ARGs such as *acrA, acrB* and *tolC* code for the important AcrAB-TolC efflux pump, which confers resistance against cephalosporins, phenicols, fluoroquinolones and penicillins.^39,40^ These resistance mechanisms appear to be stabilized in the bacterial population regulated by factors like transferability, cost-free resistance mutation, restoration of fitness cost by mutation without loss of resistance, or by genetic relatedness and resistance co-selection.^38,41^

We perceived that an increase in the frequency of ARG per sample was accompanied by a decline in ARG abundance. We hypothesized that the fitness cost involved in the carriage capacity of higher ARG copies might be responsible for a reduced abundance of each ARG.^42^ Again, with higher ARG frequency comes greater diversity in AMR determinants of different origins, mechanisms, and functionalities. Correspondingly, the positive correlation among ARGs of different antimicrobial classes signified the co-occurrence of ARGs, giving rise to the existence of MDR and extensively drug-resistant organisms in the setting.^43^

A high modularity index of the network analysis suggested the existence of key longitudinal patterns in the ARG persistence.^44^ A common trend of ARG abundance dynamics among different ARG subtypes may be attributed to a shared location on the mobile genetic element(s).^45,46^ With precautions, this principle can be applied for AMR monitoring and predicting co-occurring ARG abundance with their power functionality.^25,47^ We observed at least three distinct patterns for three ARG modularity classes, giving rise to the postulation that ARGs vary widely in their persistence.^41^ The ubiquitous distribution and consistently high abundance of *tetB* and *tetC-01* might be the reflection of common tetracycline use in livestock production.^48^ Environmental factors like temperature, pH, disinfection treatment, and host availability (bacteria or bacteriophage) significantly affect ARG persistence.^49^

Our linear regression modelling confirmed that the total ARG abundance in the treatment flock samples was contributed by factors, namely frequency, duration and interval of AMU, age of the birds and number and combination of antimicrobial classes. Each of these variables explained approximately 10% of the total ARG variance individually. Statistical modelling showed that only 10%–42% of the ARG abundance variation can be explained by direct AMU.^20^ Intense selective pressure deployed by multiple antimicrobials for a prolonged period may be the most important factor in describing ARG abundance, as revealed in multivariable analysis results.

One of the key challenges of this study was using 16S rRNA as the normalization factor to calculate the ARG abundance due to its variability in gene copy number. Other limitations include data unavailability on the origin of chicks, dose of antimicrobials administered by farmers and farm biosecurity practices, AMU variability across the farms and multiple exposure to different antimicrobials in most of the farms. These factors lead to a disparity in the microbial composition across the samples, making such a comparison difficult. Finally, the findings of this study revealed that while administering antimicrobials, multiple AMU-related factors contribute differently to developing AMR, which can help mitigate the problem. Future studies should be conducted to exploit the role of each factor in the enrichment of ARGs. Variations in the farming approaches and management practices between LMICs and developed countries should be considered critically in the study design. Measuring the health impact of consumption of farm products with and without farm-level AMU can also be investigated to confirm the success of veterinary AMR intervention strategies.

## Supplementary Material

dlaf117_Supplementary_Data

## Supplementary data

Figures S1 to S4 and Tables S1 to S8 are available as Supplementary data at *JAC-AMR*  Online.
