# Supplementary material for: High abundance of antimicrobial resistance genes in chicken flocks receiving antimicrobial treatment in Vietnamese poultry production
Source: JAC Antimicrob Resist. 2025 Jul 4;7(4):dlaf117. doi: 10.1093/jacamr/dlaf117 (PMC12231541; doi:10.1093/jacamr/dlaf117)
Supplement: dlaf117_Supplementary_Data [file dlaf117_supplementary_data.docx]

**Supplementary data**

**Manuscript title:** High abundance of antimicrobial resistance genes in chicken flocks receiving antimicrobial treatment in Vietnamese poultry production

**Table S1: Descriptive summary of the farm-level AMU**

| **Farm ID** | **AMU Frequency** | **Total AMU duration** | **Bird’s age receiving antimicrobials (days)** | **Antimicrobials administered**  **(no of class of antimicrobials)** |
| --- | --- | --- | --- | --- |
| K06 | 2 | 7 | 30, 35 | Sulfonamides, Tetracyclines, MLS_B_ (3) |
| K07 | 3 | 8 | 1, 36, 38 | Tetracyclines, Polymyxin, Beta-lactams, Quinolones (4) |
| K08 | 1 | 2 | 8 | Tetracyclines, MLS_B_ (2) |
| K09 | 1 | 2 | 87 | Tetracyclines, Polymyxins (2) |
| K11 | 1 | 3 | 3 | Beta-lactams, Aminoglycosides (2) |
| K12 | 1 | 4 | 3 | Beta-lactams, Aminoglycosides (2) |
| K13 | 2 | 7 | 1, 2 | Tetracyclines, MLS_B_, Beta-lactams, Polymyxins (4) |
| K14 | 4 | 38 | 1, 21, 35, 42 | Polymyxins, Tetracyclines, Sulfonamides, MLS_B_ (4) |
| K15 | 3 | 31 | 1, 21, 35 | Polymyxins, Tetracyclines, Sulfonamides (3) |
| K16 | 4 | 36 | 1, 21, 35, 49 | Polymyxins, Tetracyclines, Sulfonamides, Phenicols, Aminoglycosides (5) |
| K17 | 3 | 24 | 1, 13, 28 | Polymyxins, Tetracyclines, Phenicols (3) |
| K18 | 3 | 31 | 1,21,35 | Polymyxins, Tetracyclines, Sulfonamides (3) |
| K19 | 1 | 3 | 1 | Quinolones (1) |
| K20 | 2 | 19 | 1,14 | Polymyxins, Aminoglycosides, MLS_B_, Tetracyclines, Sulfonamides (5) |
| K21 | 4 | 28 | 1,14,21,61 | Sulfonamides, Tetracyclines (2) |
| K22 | 2 | 18 | 1,14 | Polymyxins, Tetracyclines (2) |
| K23 | 3 | 24 | 1,14,21 | Sulfonamides, Tetracyclines, MLS_B_, Polymyxins (4) |
| K24 | 1 | 5 | 15 | Tetracyclines (1) |
| K25 | 1 | 4 | 4 | Quinolones (1) |
| K26 | 1 | 4 | 40 | Tetracyclines (1) |

**Table S2:** List of assays used in the study with their forward, reverse primer sequences and antimicrobial class

| **Assay name** | **Forward primer** | **Reverse primer** | **Antimicrobial class (Reference)** |
| --- | --- | --- | --- |
| *16S rRNA* | CAACGCGARGAACCTTACC | ACAACACGAGCTGACGAC | 16S rRNA^1^ |
| *aac(3’)-Ii(acde)* | TGACGTATGAGATGCCGATG | GAGAATGCCGTTTGAATCGT | Aminoglycoside^1^ |
| *aac(6’)-aph(2”)* | TCCAAGAGCAATAAGGGCATA | TGCCCTCGTGTAATTCATGT | Aminoglycoside^1^ |
| *aac(6’)-Ib* | TTGCAATGCTGAATGGAGAG | TGGTCTATTCCGCGTACTCC | Aminoglycoside^1^ |
| *aac(6')-Ii* | AGACAGCTCGGCAGAAGAAG | ACCGTATTGAGGGATTGCAC | Aminoglycoside^1^ |
| *aac(6')-IIa* | GAACACTACCTGCCCAGAGC | GCGACGTACGACTGAGCATA | Aminoglycoside^2^ |
| *aadA* | GAACATAGCGTTGCCTTGGT | GCTGCGAGTTCCATAGCTTC | Aminoglycoside^2^ |
| *aadE* | TGTGCCGCAAAGAGATACTG | TTATCCCAACCTTCCACGAC | Aminoglycoside^2^ |
| *aadE-like* | GCATGATTTCCTGGCTGATT | CCACAATTCCTCTGGGACAT | Aminoglycoside^2^ |
| *aph(2”)-Ib* | ATCAAATCCCTGCGGTAGTG | CAAGGGCATCCTTTTCCTTT | Aminoglycoside^2^ |
| *aph(2”)-I(de)* | CGGAGGTGGTTTTTACAGGA | TTGCTTCGGCAGATTATTGA | Aminoglycoside^2^ |
| *aph(3’)-Ia-Ic* | ATTCTCACCGGATTCAGTCG | ATTCCGACTCGTCCAACATC | Aminoglycoside^2^ |
| *aph(3’)-III* | CCGGTATAAAGGGACCACCT | CTTTGGAACAGGCAGCTTTC | Aminoglycoside^2^ |
| *strB* | GGCGATTATAGCCGATCAAA | CGCGACTGGAGAACATGATA | Aminoglycoside^2^ |
| *sat4* | GAATGGGCAAAGCATAAAAACTTG | CCGATTTTGAAACCACAATTATGATA | Aminoglycoside^3^ |
| *spc* | TGACGAACGCAATGTGATTT | TCAGCTGCCAGATCTTTTGA | Aminoglycoside^1^ |
| *bla_ACC_* | TTGTTACGCTACGTGCAAGC | CGATTTGAAATAGCCGGTGT | Beta-Lactam^1^ |
| *bla_AMPC_* | ACCGCTAAACAGTGGAATGG | GCAAGTCGCTTGAGGATTTC | Beta-Lactam^1^ |
| *bla_CMY-1/MOX_* | GATCTGCTGCGTTTTGTGAA | CTACCGAGTAATGCCCTTGG | Beta-Lactam^1^ |
| *bla_CMY-2_* | CGATCCGGTCACGAAATACT | CCTGCCGTATAGGTGGCTAA | Beta-Lactam^1^ |
| *bla_CTX-M_* | ACTATGGCACCACCAACGAT | GGTTGAGGCTGGGTGAAGTA | Beta-Lactam^1^ |
| *bla_DHA_* | AAAGTGCGCAAAGCCAGTAT | AAGATTCCGCATCAAGCTGT | Beta-Lactam^1^ |
| *bla_GES_* | CTGCTGCAATGACGCAGTAT | TATCTCTGAGGTCGCCAGGT | Beta-Lactam^2^ |
| *bla_SHV_* | CTTTCCCATGATGAGCACCT | AGATCCTGCTGGCGATAGTG | Beta-Lactam^2^ |
| *bla_TEM_* | AAGCCATACCAAACGACGAG | TTGCCGGGAAGCTAGAGTAA | Beta-Lactam^2^ |
| *cblA* | TGCCTGCGACATCTTGATAG | CCGTCTTCTGTTTCCGAGAG | Beta-Lactam^2^ |
| *cepA* | ATGTCCTGCCCTGGTAGTTG | CTTGCCCGTCGATAATGACT | Beta-Lactam^2^ |
| *cepA_2* | TGCACCAAGACGAAAGTCTG | ACAGTGCTTCTTTGCGGAAT | Beta-Lactam^2^ |
| *cfxA* | GCGCAAATCCTCCTTTAACA | ACAATAACCGCCACACCAAT | Beta-Lactam^2^ |
| *mecA* | TCCAGGAATGCAGAAAGACC | GGCCAATTCCACATTGTTTC | Beta-Lactam^2^ |
| *bla_PER-1_* | CCAGCTGCTTTGCATGACTA | CTCTGGTCCTGTGGTGGTTT | Beta-Lactam^4^ |
| *bla_PSE_* | TTGTGACCTATTCCCCTGTAATAGAA | TGCGAAGCACGCATCATC | Beta-Lactam^3^ |
| *blaZ* | GGAGATAAAGTAACAAATCCAGTTAGATATGA | TGCTTAATTTTCCATTTGCGATAAG | Beta-Lactam^5^ |
| *fox5* | GGTTTGCCGCTGCAGTTC | GCGGCCAGGTGACCAA | Beta-Lactam^5^ |
| *femA* | CTTACTTACTGGCTGTACCTG | ATGTCGCTTGTTATGTGC | Beta-Lactam^6^ |
| *ermA* | GAGGGGTTTACCGCTTCTTT | ATCGGATCAGGAAAAGGACA | MLS_B_^2^ |
| *ermB* | GGTTGCTCTTGCACACTCAA | CTGTGGTATGGCGGGTAAGT | MLS_B_^2^ |
| *ermC* | TGAAATCGGCTCAGGAAAAG | GGTCTATTTCAATGGCAGTTACG | MLS_B_^2^ |
| *mefA_10* | CCTGCAAATGGCGATTATTT | CCAAAGACCGCATAGGGTAA | MLS_B_^2^ |
| *mefA_3* | TTACCCTATGCGGTCTTTGG | GAACCAGCTGCTGCGATAAT | MLS_B_^2^ |
| *vatA* | AACAGCTTCTGCAGCAATGA | CCTTGAAAGGGGACATTGAA | MLS_B_^2^ |
| *vatB* | TGGGAAAAAGCAACTCCATC | TTCTGACCAATCCACACATCA | MLS_B_^2^ |
| *lnu(A)-01* | TGACGCTCAACACACTCAAAAA | TTCATGCTTAAGTTCCATACGTGAA | MLS_B_^5^ |
| *macB* | GGCTGGAAGACCGTACAGAG | GTTGGTTCATCGGCAAGAAT | MLS_B_^1^ |
| *cmr* | CGGCATCGTCAGTGGAATT | CGGTTCCGAAAAAGATGGAA | Multidrug^7^ |
| *oqxA* | GACAGCGTCGCACAGAATG | GGAGACGAGGTTGGTATGGA | Multidrug^8^ |
| *oqxB* | CGAAGAAAGACCTCCCTACCC | CGCCGCCAATGAGATACA | Multidrug^8^ |
| *mfsA* | AATATGCTCTCCGGGCTTTT | TTTGCACACCGTAAAATGGA | Multidrug^2^ |
| *oprD* | ATGAAGTGGAGCGCCATTG | GGCCACGGCGAACTGA | Multidrug^3^ |
| *acrA* | GAAGGTAGCGACATCGAAGC | CTTTCGCCAGATCACCTTTC | Multidrug^1^ |
| *acrB* | CACGGTGACACAGGTTATCG | AAGGTCAGGGTGATCTGCAC | Multidrug^2^ |
| *acrF* | ACTGACACCGGTTGATGTGA | GAGCAATAATCGAGGCGTTC | Multidrug^2^ |
| *mdtF* | GGACCCGCAAAAACTCAATA | AGTTGACCACCGGAAATCTG | Multidrug^2^ |
| *mdtL* | CGGACAAACCACGAGAAAAT | GAAGGTGAGGATCACCGAAA | Multidrug^2^ |
| *mdtO* | TTGTTGGCCTCTATCCAACC | TTAAGCGCTTGATGCATTTG | Multidrug^2^ |
| *tolC* | CTGAAAGAAGCCGAAAAACG | CGTCGGTAAGTGACCATCCT | Multidrug^2^ |
| *cfr* | CAAACGAAGGGCAGGTAGAA | GACCACAAGCAGCGTCAATA | Multidrug^2^ |
| *cfr_2* | GCCGGAGCTTTTCCTCTACT | GGTGCCGAAAGTCAAAACAT | Multidrug^2^ |
| *qacA* | GACCCTTCTGGTACCCAACA | TCCCCATTTATCAGCAAAGG | Multidrug^2^ |
| *qacC* | TGGGCGGGACTAGGTTTAG | ACGAAACTACGCCGACTATGA | Multidrug^2^ |
| *qacE* | TCGGTGTTGCTTATGCAGTC | ATCAAGCTTTTGCCCATGAA | Multidrug^2^ |
| *cat* | CAATCCTCAATCGACACGAA | GATTGTGTAGCAAGGCAGCA | Phenicol^2^ |
| *cmlA1-01* | TAGGAAGCATCGGAACGTTGAT | CAGACCGAGCACGACTGTTG | Phenicol^3^ |
| *floR* | ATTGTCTTCACGGTGTCCGTTA | CCGCGATGTCGTCGAACT | Phenicol^9^ |
| *fexA* | TCGCTGTTCTTGTGTTCGTC | ACAGCCCCATCAGAGTCATC | Phenicol^10^ |
| *mcr-2* | TGTTGCTTGTGCCGATTGGA | AGATGGTATTGTTGGTTGCTG | Polymyxin^11^ |
| *mcr-1* | CGGTCAGTCCGTTTGTTC | CTTGGTCGGTCTGTAGGG | Polymyxin^12^ |
| *mcr-3* | TTGGCACTGTATTTTGCATTT | TTAACGAAATTGGCTGGAACA | Polymyxin^13^ |
| *arnA* | GAAATTCACCGTCTGGTCGT | GTGGTGCAACAGAAATCACG | Polymyxin^2^ |
| *qnrA* | ATTTCTCACGCCAGGATTTG | ACTGCAATCCTCGAAACTGG | Quinolone^2^ |
| *qnrB* | CGATCTGACCAATTCGGAGT | ACGATGCCTGGTAGTTGTCC | Quinolone^2^ |
| *qnrC* | GCAGAATTCAGGGGTGTGAT | AACTGCTCCAAAAGCTGCTC | Quinolone^2^ |
| *qnrS* | TGGAAACCTACCGTCACACA | AATCGCATCGGATAAAGGTG | Quinolone^2^ |
| *sul1* | AGGCTGGTGGTTATGCACTC | AAGAACCGCACAATCTCGTC | Sulfonamide^2^ |
| *folA* | CGAGCAGTTCCTGCCAAAG | CCCAGTCATCCGGTTCATAATC | Sulfonamide^7^ |
| *sul3* | TCCGTTCAGCGAATTGGTGCAG | TTCGTTCACGCCTTACACCAGC | Sulfonamide^14^ |
| *dfrA* | AGCACGATAGTAGCCGCAGT | AAGGTTTTGGGGAAATCGTC | Sulfonamide^2^ |
| *dfrF* | GATTGTTGCGAGGTCAAAGAA | CGCCCCATAATAACCACATT | Sulfonamide^2^ |
| *sul2* | TCATCTGCCAAACTCGTCGTTA | GTCAAAGAACGCCGCAATGT | Sulfonamide^3^ |
| *tetB* | CAAAACTTGCCCCTAACCAA | GCTTTCAGGGATCACAGGAG | Tetracycline^1^ |
| *tetM* | TTGATGCGGGAAAAACTACC | TACCTCTGTCCACGCTTCCT | Tetracycline^1^ |
| *tetO* | GCGTCAAAGGGGAATCACTA | CGGTATACTTCCGCCAAAAA | Tetracycline^1^ |
| *tetQ* | GCAAAGGAAGGCATACAAGC | AAACGCTCCAAATTCACACC | Tetracycline^1^ |
| *tetW* | GGTGCAGTTGGAGGTTGTTT | AAATGACGGAGGGTTCCTTT | Tetracycline^1^ |
| *tetX* | CGGTACGCTGGATTTACACA | CATCGGAATTGCCTTTTTGT | Tetracycline^1^ |
| *tetA-01* | GCTGTTTGTTCTGCCGGAAA | GGTTAAGTTCCTTGAACGCAAACT | Tetracycline^5^ |
| *tetC-01* | CATATCGCAATACATGCGAAAAA | AAAGCCGCGGTAAATAGCAA | Tetracycline^5^ |
| *vanA* | GTGCGGTATTGGGAAACAGT | TGCGTTTTCAGAGCCTTTTT | Vancomycin^1^ |
| *vanB* | CCTGCCTGGTTTTACATCGT | GCTGTCAATCAGTGCAGGAA | Vancomycin^1^ |
| *bacA* | AACTTCCCGTTCTGGTGCTA | CATAACGGGGATAGCGAGAA | Other^1^ |
| *bacA_1* | GGCTGCGTTACTGTCGTTTT | GGCCAATGATAAATGCATCC | Other^1^ |
| *bacA_2* | GAGGCATTGATCCTTGGTGT | AAACAATGCCGAACCGATAG | Other^1^ |
| *fosB* | TTGAGCTTGCAGGCCTATG | GCCAATATTTAAATTCGCTGTCA | Other^2^ |
| *fosX* | GATTAAGCCATATCACTTTAATTGTGAAAG | TCTCCTTCCATAATGCAAATCCA | Other^5^ |

**Table S3:** Number of unique ARG under each antimicrobial class in the treatment and control group

| **Antimicrobial class** | **Unique ARG (Control group)** | **Percentage (%)** | **Unique ARG (Treatment group)** | **Percentage (%)** |
| --- | --- | --- | --- | --- |
| Aminoglycoside | 14 | 18.9 | 14 | 18.4 |
| Beta-Lactam | 14 | 18.9 | 15 | 19.7 |
| Glycopeptide | 1 | 1.4 | 1 | 1.3 |
| MLS_B_ | 7 | 9.5 | 7 | 9.2 |
| Multidrug | 16 | 21.6 | 15 | 19.7 |
| Phenicol | 1 | 1.4 | 1 | 1.3 |
| Polymyxin | 2 | 2.7 | 3 | 3.9 |
| Quinolone | 3 | 4.1 | 3 | 3.9 |
| Sulfonamide | 6 | 8.1 | 6 | 7.9 |
| Tetracycline | 6 | 8.1 | 6 | 7.9 |
| Other | 4 | 5.4 | 5 | 6.6 |
| **Total** | **74** | **100** | **76** | **100** |

**Table S4: Antimicrobial class-wise average normalised ARG abundance at different sampling time points between treatment and control group chicken flocks**

| **Antimicrobial class** | **Mean normalized ARG abundance (log2 value) (95% CI)** | | | | | | | |
| --- | --- | --- | --- | --- | --- | --- | --- | --- |
|  | **Before** | **After** | **Day 7** | **Day 14** | **Day 30** | **Day 60** | **Day 90** | **End** |
| **All ARGs** |  |  |  |  |  |  |  |  |
| *Control* | 3.4  (3.1, 3.8) | 2.2  (2.0, 2.5) | 3.1  (2.8, 3.4) | 2.6  (2.4, 2.8) | 1.5  (1.2, 1.7) | 1.6  (1.4, 1.8) | 1.4  (1.1, 1.7) | 2.6  (2.3, 2.9) |
| *Treatment* | 3.5  (3.3, 3.7) | 3.2  (3.1, 3.5) | 2.1  (1.8, 2.3) | 2.7  (2.5, 2.9) | 2.3  (2.1, 2.5) | 2.8  (2.6, 3.1) | 3.0  (2.7, 3.2) | 3.8  (3.5, 4.1) |
| *p-value* | 0.611 | <0.001 | <0.001 | 0.552 | <0.001 | <0.001 | <0.001 | <0.001 |
| **Aminoglycoside** |  |  |  |  |  |  |  |  |
| *Control* | 4.4  (3.7, 5.1) | 2.7  (2.1, 3.3) | 3.8  (3.2, 4.4) | 3.0  (2.5, 3.5) | 1.8  (1.3, 2.2) | 2.6  (2.2, 3.1) | 2.3  (1.7, 2.8) | 3.1  (2.5, 3.7) |
| *Treatment* | 4.0  (3.5, 4.4) | 3.8  (3.4, 4.4) | 2.8  (2.4, 3.3) | 3.5  (3.1, 4.0) | 3.3  (2.9, 3.8) | 4.1  (3.7, 4.5) | 4.1  (3.6, 4.6) | 4.8  (4.2, 5.3) |
| *p-value* | 0.282 | 0.002 | 0.010 | 0.124 | <0.001 | <0.001 | <0.001 | <0.001 |
| **Beta-lactam** |  |  |  |  |  |  |  |  |
| *Control* | 1.4  (0.5, 2.3) | 0.4  (-0.2, 1.1) | 1.6  (0.9, 2.3) | 0.8  (0.3, 1.3) | -0.8  (-1.3, -0.3) | -0.7  (-1.2, -0.3) | -1.2  (-1.8, -0.6) | 0.3  (-0.5, 1.1) |
| *Treatment* | 1.8  (1.2, 2.3) | 2.0  (1.4, 2.5) | 0.5  (0.1, 1.0) | 0.7  (0.3, 1.2) | 0.3  (-0.2, 0.8) | 0.7  (0.2, 1.2) | 0.7  (-0.04, 1.3) | 1.9  (1.0, 2.7) |
| *p-value* | 0.468 | <0.001 | 0.009 | 0.928 | 0.003 | <0.001 | <0.001 | 0.010 |
| **MLS_B_** |  |  |  |  |  |  |  |  |
| *Control* | 4.5  (3.6, 5.5) | 3.5  (2.7, 4.4) | 4.5  (3.7, 5.3) | 4.5  (3.9, 5.1) | 3.3  (2.7, 3.9) | 3.2  (2.6, 3.7) | 3.3  (2.7, 4.0) | 4.0  (3.3, 4.6) |
| *Treatment* | 4.5  (3.6, 5.5) | 4.0  (3.3, 4.6) | 3.3  (2.7, 3.9) | 4.0  (3.5, 4.5) | 3.7  (3.2, 4.3) | 3.7  (3.2, 4.2) | 3.8  (3.3, 4.4) | 5.1  (4.4, 5.9) |
| *p-value* | 0.927 | 0.407 | 0.013 | 0.211 | 0.339 | 0.138 | 0.264 | 0.016 |
| **Multidrug** |  |  |  |  |  |  |  |  |
| *Control* | 3.2  (2.4, 3.9) | 2.3  (1.8, 2.9) | 2.8  (2.2, 3.3) | 2.2  (1.8, 2.6) | 1.5  (1.1, 2.0) | 0.8  (0.4, 1.3) | 0.8  (0.2, 1.3) | 2.8  (2.2, 3.3) |
| *Treatment* | 3.7  (3.2, 4.1) | 3.4  (2.9, 3.8) | 2.1  (1.7, 2.6) | 2.9  (2.4, 3.3) | 2.6  (2.1, 3.0) | 3.0  (2.6, 3.4) | 3.4  (2.9, 3.8) | 4.1  (3.5, 4.7) |
| *p-value* | 0.236 | 0.003 | 0.076 | 0.027 | 0.002 | <0.001 | <0.001 | 0.001 |
| **Phenicol** |  |  |  |  |  |  |  |  |
| *Control* | -0.3  (-5.8, 5.2) | -0.4  (-2.3, 1.6) | 0.2  (-2.8, 3.1) | 0.3  (-0.9, 1.5) | -2.5  (-4.0, -0.9) | -0.8  (-1.7, 0.2) | -0.8  (-2.4, 0.9) | 0.4  (-5.7, 6.5) |
| *Treatment* | 2.6  (1.3, 4.0) | -0.4  (-2.2, 1.5) | -2.1  (-3.4, -0.7) | -0.5  (-1.7, 0.6) | -3.0  (-4.4, -1.5) | -0.5  (-1.9, 0.9) | -0.01  (-2.1, 2.1) | 0.4  (-2.4, 3.2) |
| *p-value* | 0.094 | 0.992 | 0.121 | 0.283 | 0.606 | 0.746 | 0.529 | 0.989 |
| **Polymyxin** |  |  |  |  |  |  |  |  |
| *Control* | 1.7  (-0.2, 3.7) | 0.8  (-0.6, 2.3) | 1.4  (0.02, 2.8) | 1.2  (0.2, 2.2) | 0.7  (-0.6, 2.0) | -1.5  (-2.5, -0.5) | -1.3  (-2.5, -0.1) | 0.5  (-1.3, 2.3) |
| *Treatment* | 2.8  (1.9, 3.7) | 2.4  (1.5, 3.4) | 0.8  (-0.2, 1.8) | 1.1  (0.1, 2.1) | 0.6  (-0.6, 1.7) | 1.3  (0.4, 2.3) | 1.3  (-0.01, 2.6) | 2.6  (1.0, 4.2) |
| *p-value* | 0.233 | 0.062 | 0.445 | 0.924 | 0.901 | <0.001 | 0.004 | 0.066 |
| **Quinoline** |  |  |  |  |  |  |  |  |
| *Control* | 1.2  (-5.4, 7.8) | -1.1  (-2.5, 0.4) | -0.9  (-2.9, 1.1) | -1.9  (-3.3, -0.5) | -2.0  (-4.0, 0.1) | -3.6  (-5.5, -1.6) | -4.1  (-7.8, -0.4) | -0.8  (-3.2, 1.7) |
| *Treatment* | 0.4  (-1.4, 2.3) | -0.5  (-2.1, 1.0) | -2.3  (-3.6, -0.9) | -0.6  (-2.0, 0.7) | -0.9  (-2.1, 0.3) | -0.8  (-2.0, 0.4) | 0.04  (-1.3, 1.3) | -0.2  (-1.7, 1.3) |
| *p-value* | 0.716 | 0.607 | 0.217 | 0.230 | 0.322 | 0.022 | 0.009 | 0.683 |
| **Sulfonamide** |  |  |  |  |  |  |  |  |
| *Control* | 3.6  (2.4, 4.8) | 1.6  (0.8, 2.5) | 2.3  (1.3, 3.3) | 2.0  (1.4, 2.7) | 1.6  (0.8, 2.4) | 1.6  (0.8, 2.4) | 1.0  (0.1, 2.0) | 2.1  (0.9, 3.3) |
| *Treatment* | 3.3  (2.7, 4.0) | 3.3  (2.5, 4.1) | 2.2  (1.4, 3.1) | 2.8  (2.1, 3.6) | 2.7  (1.8, 3.5) | 3.0  (2.2, 3.8) | 3.1  (2.2, 4.0) | 3.6  (2.4, 4.8) |
| *p-value* | 0.627 | 0.005 | 0.926 | 0.115 | 0.062 | 0.015 | 0.002 | 0.069 |
| **Tetracycline** |  |  |  |  |  |  |  |  |
| *Control* | 4.0  (2.7, 5.2) | 4.3  (3.5, 5.0) | 4.8  (3.9, 5.7) | 5.0  (4.1, 5.8) | 3.1  (2.4, 3.8) | 3.5  (2.7, 4.3) | 3.4  (2.3, 4.4) | 3.5  (2.4, 4.7) |
| *Treatment* | 4.2  (3.4, 5.1) | 3.9  (3.1, 4.7) | 2.4  (1.5, 3.2) | 4.1  (3.4, 4.9) | 3.1  (2.4, 3.9) | 3.5  (2.8, 4.2) | 4.1  (3.3, 4.8) | 4.3  (3.2, 5.3) |
| *p-value* | 0.718 | 0.543 | <0.001 | 0.154 | 0.982 | 0.950 | 0.304 | 0.346 |
| **Glycopeptide** |  |  |  |  |  |  |  |  |
| *Control* | - | -1.1  (N/C) | - | - | - | - | -3.5  (N/C) | -3.6  (-12.2, 5.1) |
| *Treatment* | - | -4.2  (N/C) | - | -3.6  (-5.7, -1.4) | -3.4  (-20.1, 13.3) | -4.8  (N/C) | -2.7  (N/C) | -3.9  (-5.9, -1.9) |
| *p-value* | - | N/C | N/C | N/C | N/C | N/C | N/C | 0.680 |
| **Other** |  |  |  |  |  |  |  |  |
| *Control* | 3.5  (1.8, 5.2) | 2.3  (1.1, 3.6) | 3.4  (2.1, 4.7) | 2.4  (1.3, 3.5) | 1.9  (0.9, 3.0) | 1.9  (0.7, 3.1) | 1.3  (-0.2, 2.7) | 3.0  (1.4, 4.5) |
| *Treatment* | 4.0  (3.0, 5.1) | 3.8  (2.8, 4.8) | 2.8  (1.9, 3.7) | 2.8  (2.0, 3.7) | 2.5  (1.5, 3.5) | 3.4  (2.3, 4.4) | 2.8  (1.4, 4.2) | 5.1  (3.9, 6.3) |
| *p-value* | 0.569 | 0.069 | 0.446 | 0.523 | 0.431 | 0.070 | 0.123 | 0.029 |

N/C: Not calculated

**Table S5: Antimicrobial class-wise changes in the cumulative normalized ARG abundance over the sampling time points in the treatment flock**

| **Antimicrobial class** | **Per sample cumulative ARGs/16S rRNA abundance in treatment flock (log2 value) (95% CI)** | | | | | | | | **One-way ANOVA**  ***p*-value** |
| --- | --- | --- | --- | --- | --- | --- | --- | --- | --- |
|  | **Before** | **After** | **Day 7** | **Day 14** | **Day 30** | **Day 60** | **Day 90** | **End** |  |
| **All ARGs** | 19.0  (16.4, 21.7) | 19.8  (16.5, 23.1) | 12.2  (9.5, 14.9) | 16.3  (13.5, 19.0) | 13.6  (11.0, 16.3) | 16.8  (14.1, 19.5) | 17.8  (14.5, 21.1) | 21.8  (17.2, 26.4) | <0.001 |
| **Aminoglycoside** | 43.1  (35.2n, 51.0) | 49.8  (37.2, 62.4) | 35.8  (23.8, 47.8) | 45.1  (36.6, 53.6) | 41.2  (34.0, 48.3) | 52.0  (45.7, 58.2) | 52.4  (43.4, 61.5) | 62.4  (46.0, 78.9) | 0.015 |
| **Beta-lactam** | 11.6  (6.6, 16.5) | 18.1  (8.5, 27.7) | 4.9  (-1.3, 11.1) | 7.2  (-0.5, 14.9) | 2.9  (-3.0, 8.8) | 6.3  (1.6, 11.1) | 5.9  (-2.1, 14.0) | 14.9  (6.4, 23.3) | 0.019 |
| **MLS_B_** | 27.1  (20.6, 33.6) | 27.0  (19.8, 34.2) | 22.2  (15.4, 28.9) | 27.3  (21.3, 33.3) | 25.5  (20.7, 30.4) | 25.3  (20.8, 29.8) | 26.3  (20.6, 32.0) | 35.5  (25.5, 45.6) | 0.333 |
| **Multidrug** | 39.2  (29.2, 49.2) | 40.3  (27.3, 53.3) | 25.3  (14.4, 36.2) | 33.1  (21.9, 44.3) | 30.3  (20.5, 40.2) | 35.6  (27.8, 43.5) | 40.7  (32.6, 48.8) | 49.4  (36.0, 62.7) | 0.092 |
| **Phenicol** | 2.6  (1.3, 4.0) | -0.4  (-2.2, 1.5) | -2.1  (-3.4, -0.7) | -0.5  (-1.6, 0.6) | -3.0  (-4.4, -1.5) | -0.5  (-1.9, 0.9) | -0.01  (-2.1, 2.1) | 0.4  (-2.4, 3.2) | <0.001 |
| **Polymyxin** | 4.7  (2.7, 6.7) | 4.2  (2.1, 6.2) | 1.2  (-0.5, 2.9) | 2.1  (-0.2, 4.3) | 1.1  (-0.9, 3.1) | 2.1  (0.8, 3.3) | 2.2  (0.6, 38) | 4.5  (1.9, 7.0) | 0.016 |
| **Quinoline** | 0.6  (-1.6, 2.8) | -0.9  (-3.9, 2.1) | -3.5  (-6.1, -1.0) | -1.1  (-3.7, 1.5) | -1.2  (-2.6, 0.2) | -1.3  (-3.1, 0.5) | 0.1  (-2.1, 2.2) | -0.3  (-2.0, 1.3) | 0.197 |
| **Sulfonamide** | 15.0  (11.9, 18.2) | 16.2  (10.8, 21.7) | 10.8  (6.8, 14.8) | 14.0  (10.3, 17.8) | 12.7  (9.2, 16.1) | 14.7  (11.9, 17.6) | 15.4  (12.7, 18.1) | 18.9  (13.7, 24.2) | 0.166 |
| **Tetracycline** | 20.8  (15.6, 25.9) | 22.0  (16.0, 27.9) | 13.4  (6.7, 20.1) | 22.9  (19.5, 26.3) | 18.3  (12.9, 23.7) | 20.3  (15.8, 24.9) | 24.3  (19.0, 29.7) | 24.8  (17.8, 31.8) | 0.049 |
| **Glycopeptide** | 0  (0) | -4.2  (0) | 0  (0) | -0.2  (-0.3, -0.1) | -0.2  (-1.2, 0.8) | -0.3  (0) | -0.2  (0) | -0.4  (-0.6, -0.2) | N/C |
| **Other** | 8.4  (6.4, 10.5) | 10.4  (7.2, 13.6) | 7.3  (4.8, 9.8) | 8.5  (6.6, 10.4) | 6.8  (4.9, 8.6) | 7.8  (6.3, 9.3) | 7.3  (4.7, 9.8) | 10.6  (7.7, 13.5) | 0.175 |

N/C: Not calculated

**Table S6:** Prevalence and normalised abundance of individual ARG with approximation and significance test measures

| **Individual ARG with class** | **Prevalence (95% CI)** | | **Chi-square *p*-value** | **Normalized abundance**  **(mean log2 value, 95% CI)** | | **t-test *p*-value** |
| --- | --- | --- | --- | --- | --- | --- |
|  | **Control** | **Treatment** |  | **Control** | **Treatment** |  |
|  | **(n=126)** | **(n=135)** |  | **(n=126)** | **(n=135)** |  |
| **Aminoglycoside** |  |  |  |  |  |  |
| *aac(6')-Ii* | 43.7  (35.3-52.4) | 47.4  (39.2-55.8) | 0.619 | -1.5  (-2.5, -0.6) | -2.4  (-3.1, -1.8) | 0.123 |
| *aac(6')-IIa* | 52.4  (43.7-60.9) | 74.1  (66.1-80.8) | <0.001 | -1.8  (-2.5, -1.1) | 0.3  (-0.3,0.8) | <0.001 |
| *aph(2”)-I(de)* | 65.1  (56.4-72.9) | 73.3  (65.3-80.1) | 0.148 | -1.1  (-1.7, -0.5) | -0.1  (-0.7, 0.5) | 0.016 |
| *aph(2”)-Ib* | 71.4  (63.0-78.6) | 63.0  (54.6-70.7) | 0.15 | -0.8  (-1.4, -0.1) | -1.1  (-1.7, -0.6) | 0.373 |
| *aac(6’)-Ib* | 75.4  (67.2-82.1) | 91.1  (85.0-95.0) | 0.001 | -0.9  (-1.4, -0.3) | 1.8  (1.3, 2.3) | <0.001 |
| *aac(3’)-Ii(acde)* | 94.4  (88.8-97.5) | 100.0  (96.7-100.0) | 0.006 | 1.2  (0.6, 1.7) | 4.4  (4.0, 4.9) | <0.001 |
| *spc* | 98.4  (94.0-99.9) | 100.0  (96.7-100.0) | 0.232 | 3.0  (2.4, 3.5) | 4.4  (4.0, 4.8) | <0.001 |
| *aadA* | 99.2  (95.2-100.0) | 98.5  (94.4-100.0) | 0.602 | 3.9  (3.4, 4.5) | 5.4  (5.1, 5.8) | <0.001 |
| *aadE-like* | 99.2  (95.2-100.0) | 97.8  (93.4-99.5) | 0.623 | 5.4  (4.8, 6.0) | 4.9  (4.4, 5.4) | 0.229 |
| *aph(3’)-Ia,-Ic* | 99.2  (95.2-100.0) | 100.0  (96.7-100.0) | 0.957 | 3.5  (3.0, 4.0) | 5.4  (5.1, 5.8) | <0.001 |
| *strB* | 99.2  (95.2-100.0) | 99.3  (95.5-100.0) | 0.961 | 4.7  (4.2, 5.1) | 6.0  (5.6, 6.3) | <0.001 |
| *aac(6’)-aph(2”)* | 100  (96.4-100.0) | 100.0  (96.7-100.0) | 1.00 | 5.2  (4.7, 5.7) | 6.2  (5.8, 6.5) | <0.001 |
| *aadE* | 100  (96.4-100.0) | 100.0  (96.7-100.0) | 1.00 | 3.4  (2.9, 4.0) | 4.8  (4.4, 5.2) | <0.001 |
| *aph(3’)-III* | 100  (96.4-100.0) | 100.0  (96.7-100.0) | 1.00 | 6.1  (5.6, 6.6) | 5.9  (5.8, 6.3) | 0.547 |
| *sat4* | 100  (96.4-100.0) | 100.0  (96.7-100.0) | 1.00 | 5.4  (4.8, 5.9) | 5.2  (4.8, 5.8) | 0.579 |
| **Beta-lactam** |  |  |  |  |  |  |
| *blaPER-1* | 0  (0.0-0.3) | 3.0  (0.9-7.6) | 0.052 | N/C | -4.6  (-6.4, -2.9) | N/C |
| *blaGES* | 0.8  (0-0.4) | 0.7  (0.0-4.5) | 0.961 | -3.3  (N/C) | -5.9  (N/C) | N/C |
| *cepA2* | 8.7  (4.8-15.1) | 17.8  (12.2-25.2) | 0.3 | -1.8  (-4.1, 0.6) | -2.4  (-3.6, -1.1) | 0.618 |
| *cepA* | 19.8  (13.8-27.7) | 26.7  (19.9-34.7) | 0.193 | -1.6  (-2.9, -0.3) | -1.8  (-2.9, -0.7) | 0.809 |
| *blaCMY2* | 26.2  (19.3-34.5) | 40.7  (32.8-49.2) | 0.013 | -3.5  (-4.5, -2.5) | -2.4  (-3.0, -1.7) | 0.058 |
| *blaDHA* | 28.6  (21.4-37.0) | 77.8  (69.8-84.5) | <0.001 | -2.4  (-3.2, -1.6) | 0.8  (0.3, 1.4) | <0.001 |
| *blaSHV* | 28.6  (21.4-37.0) | 37.0  (29.4-45.4) | 0.146 | -2.2  (-3.2, -1.1) | -2.6  (-3.4, -1.8) | 0.527 |
| *cblA* | 44.4  (36.1-53.2) | 43.7  (35.6-52.1) | 0.904 | -1.4  (-2.4, -0.5) | -0.9  (-1.6, -0.1) | 0.340 |
| *blaCTXM* | 58.7  (50.0-67.0) | 77.0  (69.2-83.4) | 0.002 | -0.9  (-1.7, -0.1) | -0.2  (-0.8, 0.4) | 0.177 |
| *blaPSE* | 67.5  (58.9-75.0) | 91.1  (85.0-95.0) | <0.001 | -0.3  (-0.9, 0.4) | 3.1  (2.5, 3.7) | <0.001 |
| *cfxA* | 85.7  (78.5-90.9) | 83.0  (75.7-88.4) | 0.542 | 1.1  (0.4, 1.7) | 0.7  (0.1, 1.3) | 0.419 |
| *mecA* | 88.9  (82.1-93.4) | 91.1  (85.0-95.0) | 0.549 | -0.4  (-1.0, 0.1) | 1.1  (0.5, 1.6) | <0.001 |
| *blaZ* | 91.3  (84.9-95.2) | 88.9  (82.4-93.3) | 0.521 | 0.01  (-0.6, 0.7) | -0.5  (-1.0, 0.1) | 0.236 |
| *blaAMPC* | 94.4  (88.8-97.5) | 98.5  (94.4-100.0) | 0.072 | 1.3  (0.8, 1.9) | 2.6  (2.2, 3.1) | <0.001 |
| *blaTEM* | 98.4  (94.0-99.9) | 99.3  (95.5-100.0) | 0.521 | 2.7  (2.1, 3.3) | 4.6  (4.1, 5.0) | <0.001 |
| **Glycopeptide** |  |  |  |  |  |  |
| *vanB* | 0.3  (0.0-0.8) | 8.2  (4.5-14.1) | 0.085 | -3.0  (-5.1, -0.8) | -3.7  (-4.3, -3.1) | 0.355 |
| **MLS_B_** |  |  |  |  |  |  |
| *ermA* | 65.9  (57.2-73.6) | 78.5  (70.8-84.7) | 0.022 | 0.6  (-0.1, 1.3) | 0.6  (0.1, 1.2) | 0.936 |
| *macB* | 92.9  (86.8-96.4) | 98.5  (94.4-100.0) | 0.023 | 1.4  (0.9, 2.0) | 2.7  (2.3, 3.2) | <0.001 |
| *ermC* | 98.4  (94.0-99.9) | 98.5  (94.4-100.0) | 0.945 | 3.6  (3.1, 4.1) | 4.6  (4.2, 5.1) | 0.004 |
| *mefa10* | 98.4  (94.0-99.9) | 97.8  (93.4-99.5) | 0.709 | 2.6  (2.1, 3.1) | 2.9  (2.5, 3.3) | 0.384 |
| *mefA3* | 98.4  (94.0-99.9) | 99.3  (95.5-100.0) | 0.521 | 2.7  (2.2, 3.2) | 2.9  (2.5, 3.4) | 0.521 |
| *lnu(A)-01* | 99.2  (95.2-100.0) | 100.0  (96.7-100.0) | 0.3 | 7.1  (6.6, 7.6) | 5.7  (5.3, 6.2) | <0.001 |
| *ermB* | 100  (96.4-100.0) | 100.0  (96.7-100.0) | 1.00 | 6.9  (6.5, 7.4) | 7.0  (6.7, 7.4) | 0.773 |
| **Multidrug** |  |  |  |  |  |  |
| *qacA* | 0.8  (0-0.4) | 0 | N/C | -5.2  (N/C) | - | N/C |
| *oqxA* | 4.8  (2.0-10.2) | 8.9  (5.0-15.0) | 0.189 | -3.5  (-5.7, -1.3) | -1.7  (-2.7, -0.7) | 0.511 |
| *cmr* | 7.9  (4.2-14.2) | 43.0  (34.9-51.4) | <0.001 | -2.4  (-4.2, -0.6) | -1.6  (-2.2, -1.0) | 0.332 |
| *qacC* | 9.5  (5.4-16.0) | 4.4  (1.9-9.6) | 0.106 | -4.1  (-5.3, -2.9) | -2.6  (-4.4, -0.8) | 0.129 |
| *oqxB* | 28.6  (21.4-37.0) | 39.3  (31.4-47.7) | 0.069 | -1.7  (-2.7, -0.7) | -2.5  (-3.4, -1.7) | 0.199 |
| *cfr2* | 66.7  (58.0-74.3) | 86.7  (79.8-91.5) | <0.001 | -1.2  (-1.8, -0.6) | 2.1  (1.4, 2.7) | <0.001 |
| *acrB* | 90.5  (84.0-94.6) | 97.8  (93.4-99.5) | 0.015 | 0.4  (-0.2, 1.0) | 1.7  (1.2, 2.1) | <0.001 |
| *tolC* | 92.9  (86.8-96.4) | 97.8  (93.4-99.5) | 0.002 | 1.0  (0.4, 1.5) | 2.4  (2.0, 2.9) | <0.001 |
| *acrF* | 94.4  (88.8-97.5) | 97.8  (93.4-99.5) | 0.204 | 1.3  (0.8, 1.9) | 2.7  (2.3, 3.2) | <0.001 |
| *mdtO* | 96.8  (91.9-99.0) | 99.3  (95.5-100.0) | 0.152 | 1.9  (1.4, 2.5) | 3.3  (2.8, 3.7) | <0.001 |
| *mdtL* | 97.6  (92.9-99.5) | 99.3  (95.5-100.0) | 0.281 | 1.7  (1.1, 2.2) | 2.9  (2.5, 3.3) | <0.001 |
| *mfsA* | 97.6  (92.9-99.5) | 97.8  (93.4-99.5) | 0.932 | 2.4  (1.7, 3.0) | 1.9  (1.4, 2.4) | 0.301 |
| *acrA* | 98.4  (94.0-99.9) | 99.3  (95.5-100.0) | 0.611 | 2.6  (2.0, 3.1) | 4.1  (3.7, 4.6) | <0.001 |
| *mdtF* | 98.4  (94.0-99.9) | 99.3  (95.5-100.0) | 0.521 | 2.7  (2.1, 3.2) | 4.2  (3.7, 4.6) | <0.001 |
| *qacE* | 99.2  (95.2-100.0) | 99.3  (95.5-100.0) | 0.961 | 5.2  (4.6, 5.7) | 6.4  (6.0, 6.8) | <0.001 |
| **Phenicol** |  |  |  |  |  |  |
| *floR* | 100  (96.4-100.0) | 100.0  (96.7-100.0) | 1.00 | 3.4  (2.9, 3.9) | 6.0  (5.6, 6.4) | <0.001 |
| *cmlA1-01* | 96.8  (91.9-99.0) | 98.5  (94.4-100.0) | 0.362 | 1.3  (0.7, 1.8) | 3.8  (3.4, 4.2) | <0.001 |
| *cat* | 73.0  (64.6-80.0) | 74.1  (66.1-80.8) | 0.846 | -0.6  (-1.2, 0.1) | -1.0  (-1.6, -0.4) | 0.339 |
| **Polymyxin** |  |  |  |  |  |  |
| *arnA* | 92.9  (86.8-96.4) | 97.8  (93.4-99.5) | 0.058 | 1.4  (0.8, 1.9) | 2.7  (2.3, 3.2) | <0.001 |
| *mcr-1* | 54.0  (45.3-62.4) | 69.6  (61.4-76.8) | 0.009 | -1.6  (-2.3, -0.8) | -0.5  (-1.1, 0.03) | 0.022 |
| *mcr-3* | 0  (0.0-0.3) | 0.7  (0.0-4.5) | 0.333 | - | -2.3  (N/C) | N/C |
| **Quinolone** |  |  |  |  |  |  |
| *qnrA* | 0.8  (0-0.4) | 8.9  (5.0-15.0) | 0.0027 | -5.5  (N/C) | -2.9  (-4.2, -1.6) | N/C |
| *qnrB* | 21.4  (15.1-29.4) | 43.0  (34.9-51.4) | <0.001 | -2.3  (-3.5, -1.0) | -2.1  (-2.9, -1.4) | 0.808 |
| *qnrS* | 42.1  (33.8-50.8) | 74.8  (66.8-81.4) | <0.001 | -1.6  (-2.5, -0.7) | 0.2  (-0.4, 0.8) | <0.001 |
| **Sulfonamide** |  |  |  |  |  |  |
| *dfrA* | 20.6  (14.4-28.6) | 15.6  (10.3-22.7) | 0.2859 | -3.2  (-4.3, -2.2) | -4.2  (-5.2, -3.3) | 0.194 |
| *folA* | 68.3  (59.7-75.8) | 85.9  (79.0-90.9) | 0.0006 | -1.8  (-2.4, -1.2) | -0.9  (-1.4, -0.5) | 0.018 |
| *dfrF* | 88.9  (82.1-93.4) | 86.7  (79.8-91.5) | 0.5844 | -0.3  (-0.9, 0.3) | -0.3  (-0.8, 0.2) | 0.996 |
| *sul1* | 99.2  (95.2-100.0) | 99.3  (95.5-100.0) | 0.9609 | 4.3  (3.8, 4.9) | 5.6  (5.2, 6.0) | <0.001 |
| *sul2* | 99.2  (95.2-100.0) | 99.3  (95.5-100.0) | 0.9609 | 4.6  (4.1, 5.1) | 6.0  (5.7, 6.3) | <0.001 |
| *sul3* | 97.6  (92.9-99.5) | 99.3  (95.5-100.0) | 0.2811 | 1.7  (1.1, 2.3) | 4.5  (4.0, 4.9) | <0.001 |
| **Tetracycline** |  |  |  |  |  |  |
| *tetB* | 64.3  (55.6-72.1) | 82.2  (74.9-87.8) | 0.001 | -1.3  (-1.9, -0.6) | -1.1  (-1.5, -0.7) | 0.653 |
| *tetC-01* | 69.8  (61.3-77.2) | 88.2  (81.5-92.7) | <0.001 | -0.6  (-1.3, 0.1) | -0.5  (-1.0, -0.1) | 0.754 |
| *tetQ* | 97.6  (92.9-99.5) | 92.6  (86.8-96.1) | 0.0621 | 5.4  (4.7, 6.1) | 5.4  (4.8, 6.0) | 0.878 |
| *tetM* | 100  (96.4-100.0) | 100.0  (96.7-100.0) | 1.00 | 6.4  (6.0, 6.8) | 6.7  (6.4, 7.1) | 0.214 |
| *tetO* | 100  (96.4-100.0) | 100.0  (96.7-100.0) | 1.00 | 3.6  (3.1, 4.1) | 3.4  (2.9, 3.8) | 0.506 |
| *tetW* | 100  (96.4-100.0) | 100.0  (96.7-100.0) | 1.00 | 7.0  (6.5, 7.5) | 6.6  (6.2, 7.0) | 0.213 |
| **Other** |  |  |  |  |  |  |
| *fosB* | 0  (0.0-0.3) | 1.5  (0.0-5.6) | 0.1702 | - | -5.8  (-5.8, -5.8) | N/C |
| *bacA_1* | 22.2  (15.8-30.3) | 27.4  (20.6-35.5) | 0.3331 | -1.2  (-2.4, 0.04) | -1.42  (-2.5, -0.3) | 0.794 |
| *bacA_2* | 23.8  (17.2-32.0) | 25.9  (19.2-33.9) | 0.6928 | -2.8  (-3.6, -2.0) | -0.5  (-1.6, 0.6) | <0.001 |

N/C Not calculated

**Table S7:** The cumulative number of ARG within each modularity class of the network analysis

| **Antimicrobial class** | **ARG number in each modularity class** | | | | | |
| --- | --- | --- | --- | --- | --- | --- |
|  | **Module I** | **Module II** | **Module III** | **Module IV** | **Module V** | **Module VI** |
| **Beta-lactam** | 9 | 1 | 5 | 0 | 0 | 0 |
| **Aminoglycoside** | 5 | 0 | 1 | 2 | 4 | 0 |
| **Sulfonamide** | 3 | 0 | 2 | 0 | 1 | 0 |
| **Polymyxin** | 1 | 0 | 1 | 0 | 0 | 0 |
| **Quinolone** | 0 | 0 | 0 | 0 | 1 | 0 |
| **MLS_B_** | 2 | 0 | 1 | 0 | 3 | 0 |
| **Multidrug** | 6 | 0 | 7 | 0 | 1 | 0 |
| **Tetracycline** | 0 | 0 | 0 | 0 | 3 | 2 |
| **Phenicol** | 0 | 0 | 0 | 0 | 1 | 0 |
| **Other** | 1 | 1 | 0 | 0 | 1 | 0 |
| **Total** | **27** | **2** | **17** | **2** | **15** | **2** |

**Table S8:** Variables excluded from the multivariable linear regression forward model with their coefficient values

| **Model with excluded variables** | **Coefficient (B)** | **t-value** | ***p-*value** |
| --- | --- | --- | --- |
| **Model 1** |  |  |  |
| *Single AMU* | 0.039 | 0.429 | 0.668 |
| *2 times AMU* | 0.175 | 2.704 | 0.007 |
| *4 times AMU* | 0.14 | 2.153 | 0.032 |
| *AMU less than 10 days* | -0.014 | -0.17 | 0.865 |
| *AMU for 11 to 30 days* | 0.199 | 3.072 | 0.002 |
| *AMU for > 30 days* | 0.09 | 1.374 | 0.171 |
| *AMU in the first week of age* | 0.108 | 1.402 | 0.162 |
| *AMU in 4 weeks age* | 0.176 | 2.717 | 0.007 |
| *AMU in > 4 weeks* | 0.015 | 0.23 | 0.818 |
| *2 antimicrobial classes* | 0.142 | 2.171 | 0.031 |
| *5 antimicrobial classes* | 0.129 | 1.981 | 0.049 |
| *Quinolone* | 0.037 | 0.409 | 0.683 |
| *Tetracycline* | -0.037 | -0.409 | 0.683 |
| *Tetracycline + MLS_B_* | 0.106 | 1.628 | 0.105 |
| *Tetracycline + Polymyxin* | 0.077 | 1.176 | 0.241 |
| *Tetracycline + Sulfonamide* | 0.079 | 1.214 | 0.226 |
| *Tetracycline + Sulfonamide + Polymyxin + MLS_B_* | 0.082 | 1.254 | 0.211 |
| *Tetracycline + Sulfonamide + Polymyxin + Aminoglycoside + MLS_B_* | 0.145 | 2.237 | 0.026 |
| **Model 2** |  |  |  |
| *Single AMU* | 0.08 | 0.878 | 0.381 |
| *2 times AMU* | 0.115 | 1.652 | 0.1 |
| *4 times AMU* | 0.111 | 1.702 | 0.09 |
| *AMU less than 10 days* | 0.034 | 0.413 | 0.68 |
| *AMU for > 30 days* | 0.123 | 1.895 | 0.059 |
| *AMU in the first week of age* | 0.141 | 1.849 | 0.066 |
| *AMU in 4 weeks age* | 0.008 | 0.058 | 0.954 |
| *AMU in > 4 weeks* | 0.017 | 0.266 | 0.79 |
| *2 antimicrobial classes* | 0.095 | 1.423 | 0.156 |
| *5 antimicrobial classes* | 0.085 | 1.281 | 0.201 |
| *Quinolone* | 0.037 | 0.417 | 0.677 |
| *Tetracycline* | -0.037 | -0.417 | 0.677 |
| *Tetracycline + MLS_B_* | 0.121 | 1.889 | 0.06 |
| *Tetracycline + Polymyxin* | 0.026 | 0.385 | 0.7 |
| *Tetracycline + Sulfonamide* | -0.004 | -0.058 | 0.954 |
| *Tetracycline + Sulfonamide + Polymyxin + MLS_B_* | 0.035 | 0.526 | 0.599 |
| *Tetracycline + Sulfonamide + Polymyxin + Aminoglycoside + MLS_B_* | 0.075 | 1.069 | 0.286 |

**
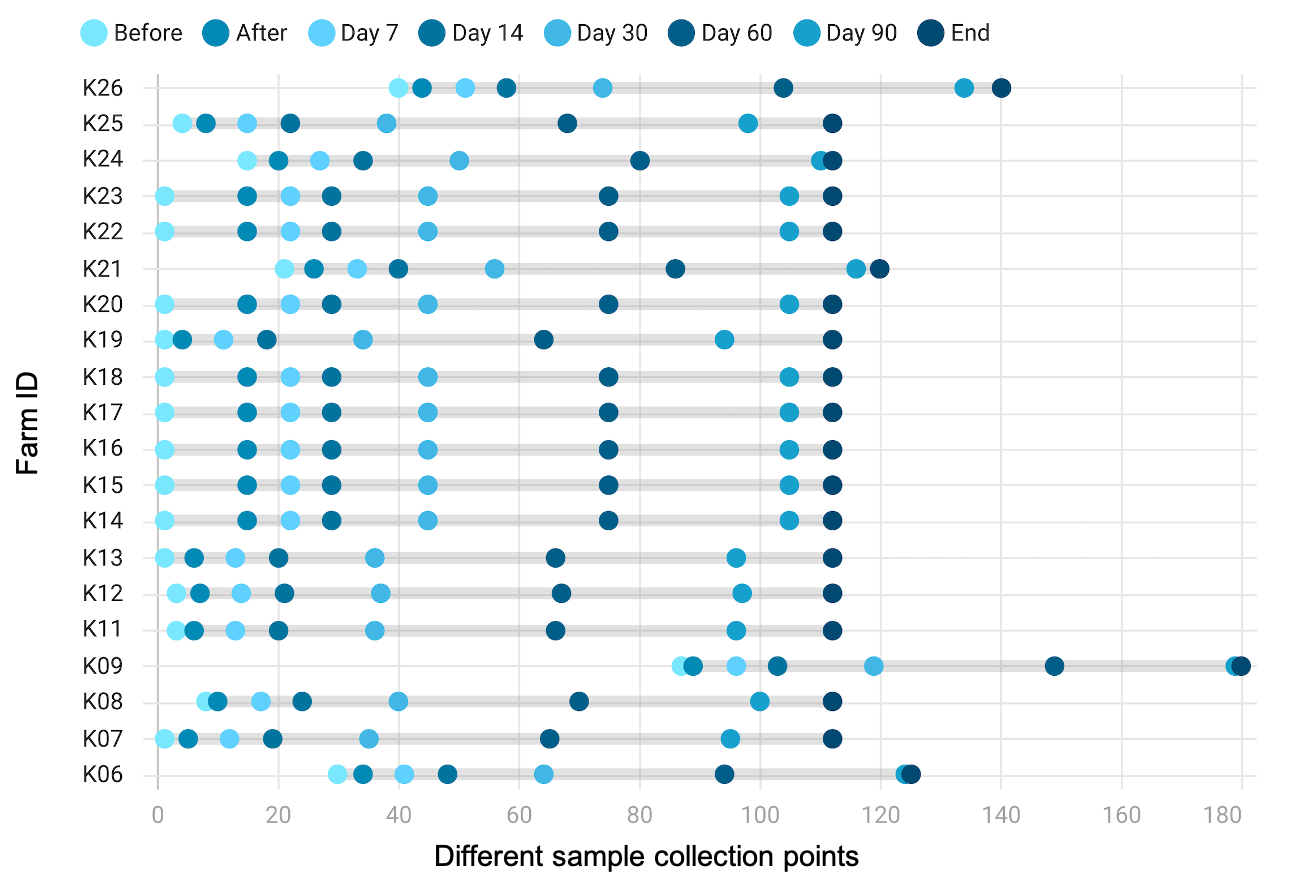
**

**Figure S1: Different sample collection time points for the recruited study farms.**

The “before” sampling was conducted prior to the first dose of antimicrobial administration to the flock. The “after” sampling denotes the end of the first round of antimicrobial treatment. The subsequent sampling points (Day 7, Day 14, Day 30, Day 60, Day 90) were determined in relation to the end of the first round of antimicrobial treatment. The “end” point marks the last sampling before depopulation of the flock. The distance between the “before” and “after” sampling represents the duration of the first round of antimicrobial to the flock in days.


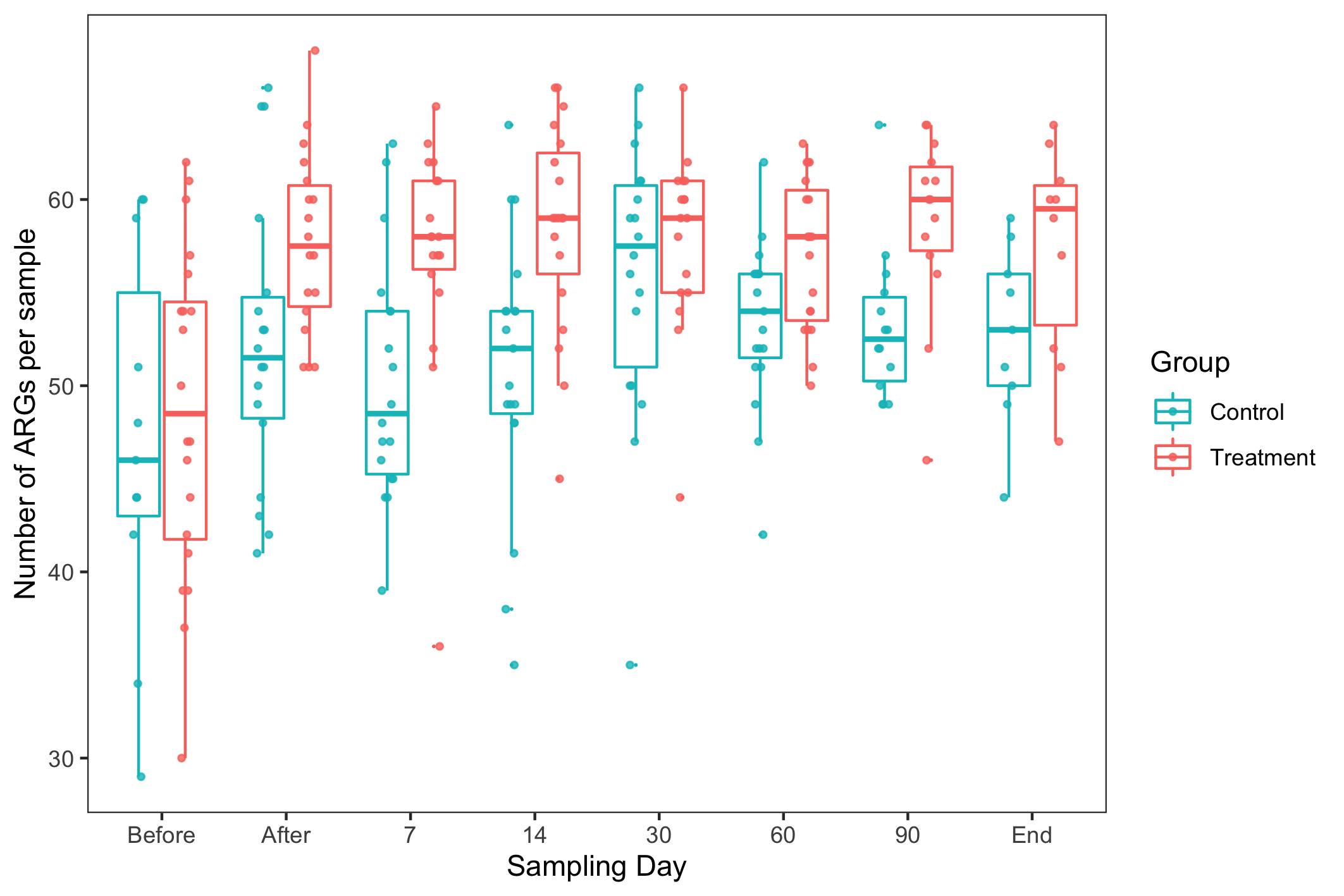


**Figure S2. Average ARG frequencies in the control and treatment flocks at different sampling time points**

Each dot represents the ARG frequency of a sample. Box plot with error bars illustrates that treatment group samples had higher ARGs/sample compared to control at all sampling time points. Here ARGs of samples accommodate resistance determinants associated with different classes of antimicrobials.

**
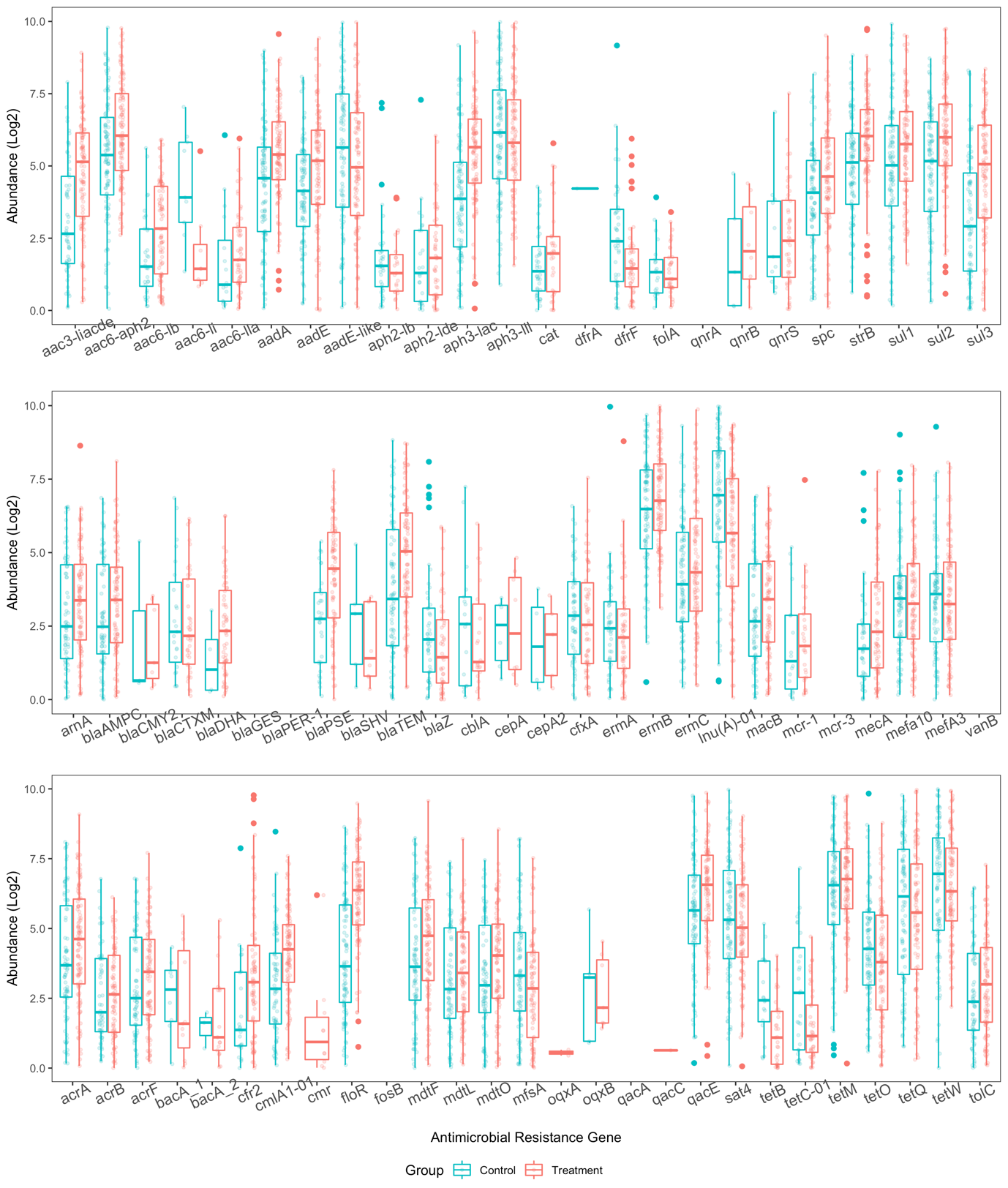
**

**Figure S3. Individual ARG abundance in treatment and control groups**

Here, for each ARG, normalised abundance data were grouped into control and treatment, irrespective of sampling time point. Each dot represents the respective ARG normalised abundance value in a sample. Box-and-whisker depicts the distribution of the data with the mean value marked. Data before antimicrobial administration was excluded from the calculation to observe the overall impact of antimicrobial exposure on the individual ARG abundance.


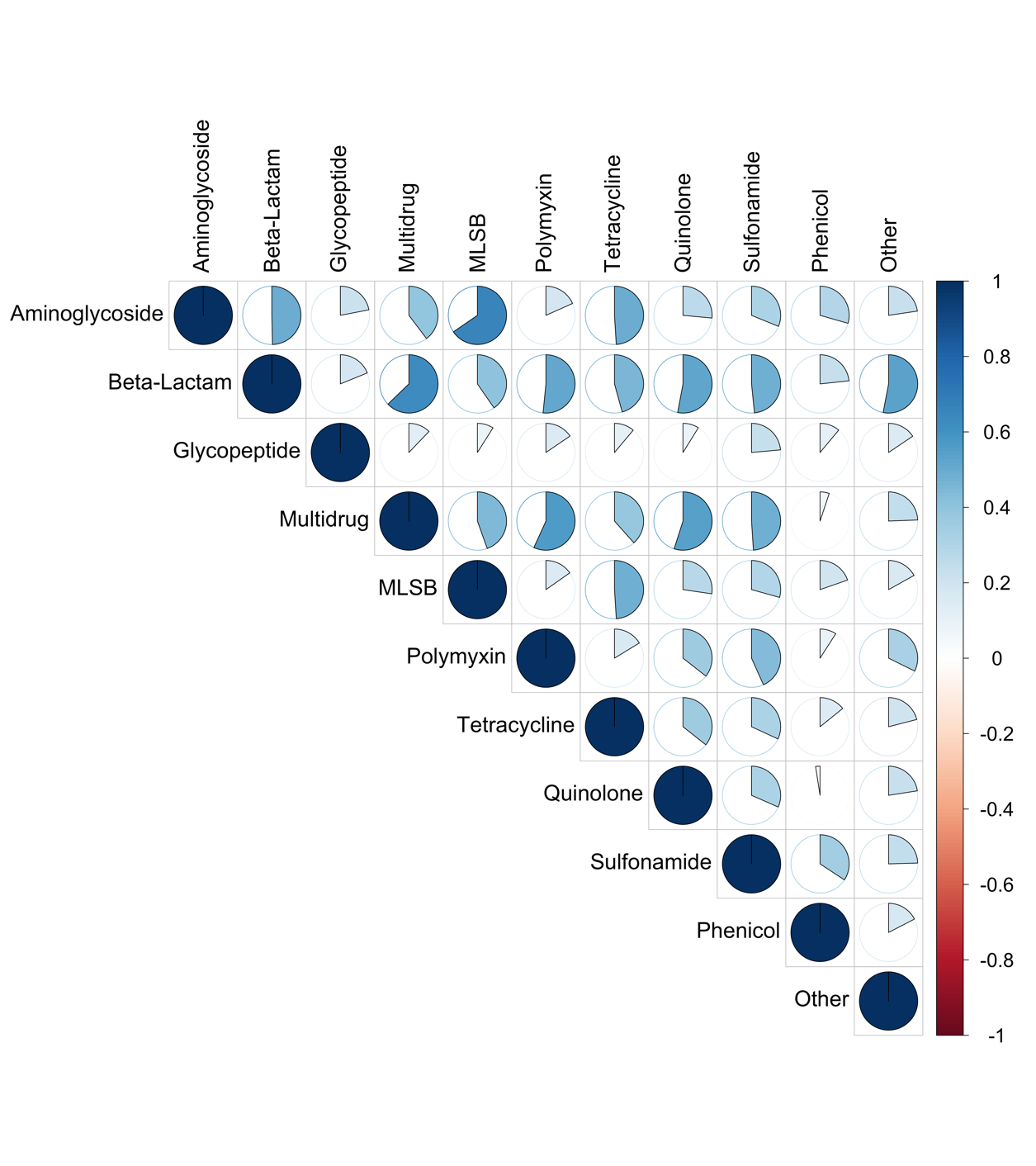


**Figure S4: Correlation matrix among ARGs conferring resistance to different antimicrobial classes**

The scale shows that blue is a positive correlation (r= 1) and red is a negative correlation (r= -1) among the ARG frequency of different antimicrobial classes. The size of the pie chart and the intensity of the colour correspond to the numerical value of the correlation coefficient (r).

**References**

**1**. Buelow E, Bayjanov JR, Majoor E *et al.* Limited influence of hospital wastewater on the microbiome and resistome of wastewater in a community sewerage system. *FEMS Microbiol Ecol* 2018; **94**: p.fiy087. <https://doi.org/10.1093/femsec/fiy087>

**2**. Gonzalez T de JB. Interplay between gut microbiota and antibiotics. 2016. Available at: https://research.wur.nl/en/publications/interplay-between-gut-microbiota-and-antibiotics. Accessed June 7, 2025

**3**. Majlander J, Anttila V-J, Nurmi W *et al*. Routine wastewater-based monitoring of antibiotic resistance in two Finnish hospitals: focus on carbapenem resistance genes and genes associated with bacteria causing hospital-acquired infections. *J Hosp Infect* 2021; **117**: 157–64. <https://doi.org/10.1016/j.jhin.2021.09.008>

**4**. Sarraf NQA, Raouf DM. Moleculer study of *Acenitobacter bumannii* associated with corona virus disease. *Intl J Health Sci* 2022; **6**: 3065–74. https://doi.org/10.53730/ijhs.v6nS1.5296

**5**. Wang H-T, Chi Q-Q, Zhu D *et al.* Arsenic and Sulfamethoxazole Increase the Incidence of Antibiotic Resistance Genes in the Gut of Earthworm. *Environ Sci Technol* 2019; **53**: 10445–53. <https://doi.org/10.1021/acs.est.9b02277>

**6**. Vannuffel P, Laterre P-F, Bouyer M *et al.* Rapid and Specific Molecular Identification of Methicillin-Resistant *Staphylococcus aureus* in Endotracheal Aspirates from Mechanically Ventilated Patients. *J Clin Microbiol* 1998; **36**: 2366–8. <https://doi.org/10.1128/jcm.36.8.2366-2368.1998>

**7**. Zhu D, Xiang Q, Yang X-R *et al.* Trophic Transfer of Antibiotic Resistance Genes in a Soil Detritus Food Chain. *Environ Sci Technol* 2019; **53**: 7770–81. https://doi.org/10.1021/acs.est.9b00214

**8**. Kao C-Y, Wu H-M, Lin W-H *et al.* Plasmid-mediated quinolone resistance determinants in quinolone-resistant *Escherichia coli* isolated from patients with bacteremia in a university hospital in Taiwan, 2001–2015. *Sci Rep* 2016; **6**: 32281. https://doi.org/10.1038/srep32281

**9**. Hayatgheib N, Calvez S, Fournel C *et al.* Antimicrobial Susceptibility Profiles and Resistance Genes in Genus *Aeromonas* spp. Isolated from the Environment and Rainbow Trout of Two Fish Farms in France. *Microorganisms* 2021; **9**: 1201. <https://doi.org/10.3390/microorganisms9061201>

**10**. Zhao Q, Wang Y, Wang S *et al.* Prevalence and Abundance of Florfenicol and Linezolid Resistance Genes in Soils Adjacent to Swine Feedlots. *Sci Rep* 2016; **6**: 32192. https://doi.org/10.1038/srep32192

**11**. Xavier BB, Lammens C, Ruhal R *et al.* Identification of a novel plasmid-mediated colistin-resistance gene, *mcr-2*, in *Escherichia coli*, Belgium, June 2016. *Euro surveill* 2016; **21**: 30280. https://doi.org/[10.2807/1560-7917.ES.2016.21.27.30280](https://doi.org/10.2807/1560-7917.es.2016.21.27.30280)

**12**. Liu Y-Y, Wang Y, Walsh TR *et al.* Emergence of plasmid-mediated colistin resistance mechanism MCR-1 in animals and human beings in China: a microbiological and molecular biological study. *Lancet Infect Dis* 2016; **16**: 161–8. https:/doi.org/[10.1016/S1473-3099(15)00424-7](https://doi.org/10.1016/s1473-3099(15)00424-7)

**13**. Yin W, Li H, Shen Y *et al.* Novel Plasmid-Mediated Colistin Resistance Gene *mcr-3* in *Escherichia coli*. *mBio* 2017; **8**: 10.1128. <https://doi.org/10.1128/mbio.00543-17>

**14**. Jiang L, Hu X, Xu T *et al.* Prevalence of antibiotic resistance genes and their relationship with antibiotics in the Huangpu River and the drinking water sources, Shanghai, China. *Sci Total Environ* 2013; **458–460**: 267–72. <https://doi.org/10.1016/j.scitotenv.2013.04.038>
